# Association of Long-Acting Injectable Antipsychotics and Oral Antipsychotics With Disease Relapse, Health Care Use, and Adverse Events Among People With Schizophrenia

**DOI:** 10.1001/jamanetworkopen.2022.24163

**Published:** 2022-07-28

**Authors:** Yue Wei, Vincent K. C. Yan, Wei Kang, Ian C. K. Wong, David J. Castle, Le Gao, Celine S. L. Chui, Kenneth K. C. Man, Joseph F. Hayes, Wing Chung Chang, Esther W. Chan

**Affiliations:** 1 Centre for Safe Medication Practice and Research, Department of Pharmacology and Pharmacy, The University of Hong Kong, Hong Kong Special Administration Region (SAR), China; 2 Laboratory of Data Discovery for Health, Hong Kong Science and Technology Park, Hong Kong SAR, China; 3 Research Department of Practice and Policy, University College London School of Pharmacy, London, United Kingdom; 4 Centre for Addiction and Mental Health, University of Toronto, Toronto, Ontario, Canada; 5 Department of Psychiatry, University of Toronto, Toronto, Ontario, Canada; 6 School of Nursing, Li Ka Shing Faculty of Medicine, The University of Hong Kong, Hong Kong SAR, China; 7 School of Public Health, Li Ka Shing Faculty of Medicine, The University of Hong Kong, Hong Kong SAR, China; 8 Division of Psychiatry, University College London, London, United Kingdom; 9 Department of Psychiatry, Queen Mary Hospital, The University of Hong Kong, Hong Kong SAR, China; 10 State Key Laboratory of Brain and Cognitive Sciences, The University of Hong Kong, Hong Kong SAR, China; 11 The University of Hong Kong Shenzhen Institute of Research and Innovation, Shenzhen, Guangdong, China

## Abstract

**Importance:**

Evidence for improved clinical outcomes with long-acting injectable antipsychotics (LAIAs) vs oral antipsychotics (OAs) is limited in Asian populations and special patient groups, including older people (>65 years), people with substance use, and early initiators of LAIAs.

**Objective:**

To compare the risk of disease relapse, health care use, and adverse events associated with the use of LAIAs vs OAs among people in Hong Kong with schizophrenia.

**Design, Setting, and Participants:**

In this self-controlled case series study, individuals with a diagnosis of schizophrenia who were prescribed LAIAs and OAs between January 1, 2004, and December 31, 2019, were identified from the Clinical Database Analysis and Reporting System of the Hong Kong Hospital Authority. Data analysis was conducted from May to August in 2021.

**Exposures:**

Use of LAIAs vs OAs.

**Main Outcomes and Measures:**

Risk of disease relapse (hospitalizations for psychiatric disorders, hospitalizations for schizophrenia, and suicide attempts), health care use (all-cause emergency department visits and hospitalizations), and adverse events (hospitalizations for somatic disorders, hospitalizations for cardiovascular diseases, and extrapyramidal symptoms) between the period in which patients were treated with LAIAs and the period in which patients were treated with OAs were compared using Poisson regression.

**Results:**

Of the 70 396 individuals with schizophrenia (37 200 women [52.8%]; mean [SD] age, 44.2 [15.8] years), 23 719 (33.7%) were prescribed both LAIAs and OAs. Compared with OAs, LAIAs were associated with a lower risk of hospitalizations for any cause (n = 20 973; incidence rate ratio [IRR], 0.63 [95% CI, 0.61-0.65]), hospitalizations for psychiatric disorders (n = 19 283; IRR, 0.52 [95% CI, 0.50-0.53]), hospitalizations for schizophrenia (n = 18 385; IRR, 0.53 [95% CI, 0.51-0.55]), and incident suicide attempts (n = 1453; IRR, 0.56 [95% CI, 0.44-0.71]). During full treatment with LAIAs, there was a reduction in hospitalizations for somatic disorders (n = 15 396; IRR, 0.88 [95% CI, 0.85-0.91]), hospitalizations for cardiovascular diseases (n = 3710; IRR, 0.88 [95% CI, 0.81-0.96]), and extrapyramidal symptoms (n = 22 182; IRR, 0.86 [95% CI, 0.82-0.91]) compared with full treatment with OAs. No significant difference was found for emergency department visits. Similar associations were observed during the subsequent treatment periods (beyond 90 days) and among older people and those with substance use, except for an increased risk of extrapyramidal symptoms among older people when initiating LAIAs (first 90 days). Compared with late initiators, early LAIA initiators had a greater reduction in these outcome events.

**Conclusions and Relevance:**

This self-controlled case series study of people in Hong Kong with schizophrenia suggests that LAIAs were associated with a lower risk of disease relapse and hospitalization than OAs, without an increased risk of adverse events. Clinicians should more broadly consider the long-term use of LAIAs for Chinese people with schizophrenia, especially early in the course of illness.

## Key Points

**Question:**

Are long-acting injectable antipsychotics (LAIAs) associated with a lower risk of disease relapse, health care use, and adverse events compared with oral antipsychotics among people in Hong Kong with schizophrenia?

**Findings:**

In this 16-year, population-based, self-controlled case series study of 70 396 individuals with a diagnosis of schizophrenia, 23 719 were prescribed LAIAs and oral antipsychotics. There were 48% fewer psychiatric hospitalizations, 47% fewer hospitalizations for schizophrenia, 44% fewer suicide attempts, and 37% fewer all-cause hospitalizations during full treatment periods with LAIAs alone, without an increased risk of adverse events; this association was also observed when excluding the first 90 days of treatment.

**Meaning:**

This study suggests that clinicians should more broadly consider the long-term use of LAIAs for people with schizophrenia.

## Teaser

This case series compares the risk of disease relapse, health care use, and adverse events associated with the use long-acting injectable antipsychotics vs oral antipsychotics among people in Hong Kong with schizophrenia.

## Introduction

Schizophrenia is a serious and often disabling mental disorder characterized by chronic or recurrent psychotic symptoms and associated functional decline.^1^ It is a global issue that affects individuals, families, and societies and is listed in the top 20 causes of global burdens of disease.^2^ For decades, antipsychotics have been prescribed to manage schizophrenia.^3^ However, nonadherence to medication remains a major challenge, as people with schizophrenia often lack social support, display poor insight, and may experience significant antipsychotic-related adverse events and stigma.^4^ Long-acting injectable antipsychotics (LAIAs) were developed specifically to improve medication adherence, with an administration interval from 2 to 12 weeks.^5^ Although major clinical guidelines recommend LAIAs for people with poor medication adherence, they remain underused because of various concerns, especially among the Chinese population and special patient groups, including older people (>65 years), people with substance use, and early LAIAs initiators.^6^

Current clinical guidelines on the use of LAIAs are derived mainly from randomized clinical trials in which strict inclusion criteria limit generalizability.^7,8,9^ Because most randomized clinical trials are of relatively short duration, data from long-term observational studies are important to establish the safety and effectiveness of LAIAs, as people with schizophrenia often require lifelong antipsychotic treatment. In addition, most published studies of LAIAs are based on Western populations,^8,10,11^ and cannot be generalized to Asian populations, for whom medication preferences, attitudes, and the potential to experience adverse effects of antipsychotics might differ. The few published observational studies of LAIAs from Asia were either from a single medical center with an unrepresentative sample^12,13,14,15^ or used an insurance claims database in which confounders could not be well adjusted for.^16,17,18,19,20^ In addition, few studies reported data on outcomes other than hospitalizations.^12,13,14,15,16,17,18^ Misclassification of exposure is also a major issue in these studies because both people treated with LAIAs alone and those treated with concurrent LAIAs and oral anticoagulants (OAs) were categorized as LAIAs users and compared with people treated with OAs alone.^18,21^

Currently, barriers still exist in the clinical use of LAIAs. A recent study found that, even for people who had commenced treatment with LAIAs, many ceased treatment because of concerns about adverse effects or a clinician’s advice that LAIAs were no longer required.^22^ Therefore, it is important to understand the effectiveness and safety of LAIAs during longer-term treatment.

There is no specific clinical recommendation on the use of LAIAs for special patient groups worldwide. Polypharmacy and pharmacokinetic changes associated with aging may increase the risk of adverse events among older people. Thus, establishing long-term safety data on LAIAs remains essential for this population.^23^ Nonadherence among people with substance use may be a particularly challenging issue, and determining the place of LAIA treatment in their management remains a pressing clinical concern.^24,25^ In addition, although early initiation has been advocated by experts and a recent 2-year randomized clinical trial,^8,26^ their long-term outcomes have not been rigorously tested.

To address these knowledge gaps, we conducted a study to compare the risk of disease relapse, health care use, and adverse events associated with the use of LAIAs vs OAs in a large population-based cohort of individuals with schizophrenia, and we performed subanalyses of special patient groups, including older people, people with substance use, and early LAIAs initiators. The findings could help clinicians and patients make better-informed decisions on the use of LAIAs.

## Method

### Data Source

We used data from the Clinical Data Analysis and Reporting System (CDARS), an electronic health records database of the Hong Kong Hospital Authority, a statutory body responsible for managing Hong Kong’s public hospitals and serving a population of more than 7.4 million people.^27^ Data from CDARS includes anonymized patient numbers, patient demographic characteristics, diagnoses, procedures, prescriptions, laboratory tests, and outpatient, inpatient, and emergency department (ED) admission records starting from 1993. Diagnostic ascertainment derived from CDARS has been used for patient identification and outcome identification in several high-quality pharmacoepidemiology studies for psychiatric disorders.^28,29,30,31,32,33^ The present study was approved by the institutional review board of the University of Hong Kong/Hospital Authority Hong Kong West Cluster. No informed consent was required because this was a register-based study using anonymized data. The data were reported using the Strengthening the Reporting of Observational Studies in Epidemiology (STROBE) reporting guideline.

### Self-controlled Case Series Design

The self-controlled case series (SCCS) study design is a within-individual comparison based on a case-only approach.^34^ Incidence rate ratios (IRRs) are derived by comparing the rate of outcomes between exposed and unexposed or reference periods for the same individual. Therefore, only people with both the exposure and the outcome are eligible. A major advantage of SCCS design is that it controls for potential measured and unmeasured time-invariant confounders that vary between individuals (eg, genetic factors).^34^ The SCCS design has been used to evaluate the association between adverse outcomes and psychotropic medications.^31,32,33^

### Case Identification

We identified the initial cohort of people with a diagnosis of schizophrenia in inpatient, outpatient, and/or ED settings in Hong Kong public health facilities before December 31, 2019, using the *International Classification of Diseases, Ninth Revision, Clinical Modification* diagnosis code 295. Cases were defined as those prescribed at least 1 OA (reference period, identified from *British National Formulary* chapter 4.2.1 “Antipsychotic drugs”)^35^ and LAIA (exposed period, *British National Formulary* chapter 4.2.2 “Antipsychotic depot injections”) and with at least 1 outcome event during the observation period. Therefore, the cases included in the SCCS analyses were different for each outcome. Individual observation period began on January 1, 2004, or the first schizophrenia diagnosis (whichever came later) and ended on December 31, 2019, or death (whichever came first).

### Exposures and Outcomes

**Figure 1.**
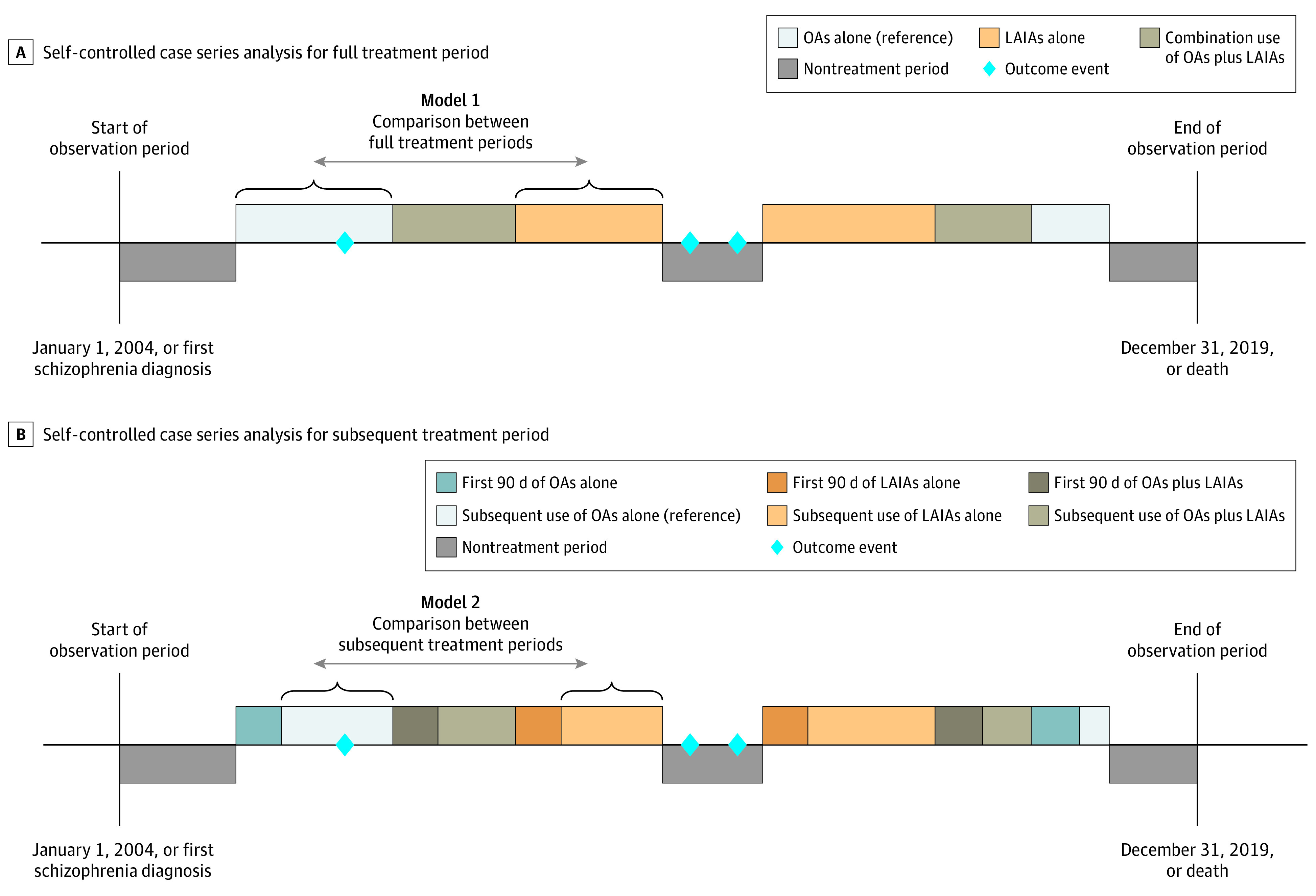
Illustration of Self-controlled Case Series Study of the Use of Long-Acting Injectable Antipsychotics (LAIAs) vs Oral Antipsychotics (OAs) Only 2 examples of the potential sequence of the medication regimen received are highlighted, for illustration purposes.

The observation period was divided into 4 periods: nontreatment period, use of OAs alone, use of LAIAs alone, and combination use of OAs and LAIAs (Figure 1A). The exact dates on which the patients were exposed to antipsychotics were derived from the start and end dates of each prescription. A washout period of 5 half-lives of each antipsychotic (eTable 1 in the Supplement) was added to account for any residual treatment effects. We further divided each antipsychotic treatment period into the first 90 days of treatment and subsequent treatment (beyond the first 90 days of each treatment) (Figure 1B). The subsequent treatment period was designed to assess the association during maintenance treatment and minimize the possibility that a recent outcome event might be associated with the likelihood of being prescribed antipsychotics and the preference of a particular antipsychotic formulation, which may introduce bias.

The primary outcomes included disease relapses (defined as hospitalizations for psychiatric disorders, hospitalizations for schizophrenia, and incident suicide attempt) and health care use (all-cause ED visits and hospitalizations). Secondary outcomes included hospitalizations for somatic disorders, hospitalizations for cardiovascular diseases, and extrapyramidal symptoms (EPS) to assess safety profiles. Detailed definitions of outcomes are described in eTable 2 in the Supplement.

### Statistical Analysis

We directly compared the risk of outcome events between the full treatment period of LAIAs alone and the full treatment period of OAs alone (Figure 1A), using standard SCCS study methods. After excluding the first 90 days of each treatment, we further assessed the association during subsequent use by comparing the outcome events between the subsequent treatment period (beyond 90 days) of LAIAs alone and the subsequent treatment period of OAs alone (Figure 1B).

We calculated adjusted IRRs and the corresponding 95% CIs using conditional Poisson regression by adjusting for time-varying factors that are potentially associated with antipsychotic prescribing and the outcomes studied, including age (1-year bands) and season (cut by the first date of March, May, September, and November). Periods of inpatient hospitalization (not including the day of admission) were excluded for the analyses related to ED visits and hospitalizations because hospitalized patients cannot attend the ED or be hospitalized elsewhere during their inpatient stay, and hence were competing events. An extension of the SCCS method was used to assess the association with suicide attempt because it may carry high short-term mortality, which could violate the SCCS assumption that the occurrence of an event should not be associated with the subsequent period of observation.^34^

### Subgroup Analyses and Additional Analyses

Subgroup analyses and sensitivity analyses were performed to test the validity of the main analyses and other SCCS assumptions (eMethods in the Supplement). An indirect comparison was conducted by comparing different windows of antipsychotic treatment with the preexposure period (30 days before any antipsychotic treatment) or baseline (nontreatment period except preexposure period) (eFigure 1 in the Supplement). Upper respiratory tract infection was used as a negative control to validate our results. E-value was calculated to assess the potential associations of any unmeasured confounding.^36^ Detailed descriptions of the analyses are presented in the eMethods in the Supplement. All *P* values were from 2-sided tests and results were deemed statistically significant at *P* < .05. All analyses were performed using R software, version 4.0.4 (R Group for Statistical Computing).

## Results

### Patient Characteristics

The CDARS contained data on 70 396 individuals (37 200 women [52.8%]; mean [SD] age, 44.2 [15.8] years) with a diagnosis of schizophrenia in Hong Kong public hospitals before December 31, 2019; 23 719 (33.7%) were prescribed both OAs and LAIAs (Table 1; eFigure 2 in the Supplement), among whom 3650 (15.4%) died during the observation period. The mean (SD) age at the start of the observation period of individuals prescribed both OAs and LAIAs was 41.7 (12.8) years. The mean (SD) duration of follow-up was 12.5 (4.7) years. The mean (SD) duration of OA exposure alone was 5.0 (5.1) years, of LAIA exposure alone was 1.4 (2.8) years, and of OA plus LAIA exposures was 4.4 (5.1) years. During the observation period, 22 013 individuals (92.8%) had at least 1 ED visit, 20 973 (88.4%) had at least 1 hospitalization, 19 283 (81.3%) were hospitalized for any psychiatric disorders, 18 385 (77.5%) were hospitalized for schizophrenia, 1453 (6.1%) had an incident suicide attempt, 15 396 (64.9%) were hospitalized for somatic disorders, 3710 (15.6%) were hospitalized for cardiovascular diseases, and 22 182 (93.5%) had EPS (Table 1).

**Table 1.** Characteristics of Patients

Characteristic	Overall (N = 70 396)	Individuals prescribed both OAs and LAIAs during observation period (n = 23 719)	Outcome events among individuals prescribed both OAs and LAIAs, No. (%)^a^							
			Primary outcomes					Secondary outcomes		
			All-cause ED visits (n = 22 013)	All-cause hospitalizations (n = 20 973)	Hospitalizations for psychiatric disorders (n = 19 283)	Hospitalizations for schizophrenia (n = 18 385)	Incident suicide attempt (n = 1453)	Hospitalizations for somatic disorders (n = 15 396)	Hospitalizations for cardiovascular diseases (n = 3710)	Extrapyramidal symptoms (n = 22 182)
Sex, No. (%)										
Female	37 200 (52.8)	11 397 (48.1)	10 551 (47.9)	10 110 (48.2)	9317 (48.3)	8859 (48.2)	613 (42.2)	7420 (48.2)	1653 (44.6)	10 646 (48.0)
Male	33 196 (47.2)	12 322 (51.9)	11 462 (52.1)	10 863 (51.8)	9966 (51.7)	9526 (51.8)	840 (57.8)	7976 (51.8)	2057 (55.4)	11 536 (52.0)
Age, mean (SD), y										
At first schizophrenia diagnosis	42.0 (15.7)	39.0 (12.7)	38.9 (12.8)	38.9 (12.9)	38.5 (12.9)	38.6 (12.9)	33.3 (10.9)	40.6 (13.3)	47.4 (12.9)	38.7 (12.5)
At cohort entry	44.2 (15.8)	41.7 (12.8)	41.7 (12.9)	41.8 (13.0)	41.3 (13.0)	41.4 (13.0)	36.1 (11.1)	43.7 (13.3)	51.0 (12.5)	41.5 (12.6)
At time of event	NA	NA	48.1 (13.7)	50.9 (15.0)	47.7 (14.5)	48.4 (14.5)	41.2 (12.1)	56.0 (14.4)	62.4 (12.4)	43.0 (12.8)
Death										
No. (%) of patients who died during observation period	12 493 (17.7)	3650 (15.4)	3512 (16.0)	3379 (16.1)	3003 (15.6)	2867 (15.6)	236 (16.2)	3014 (19.6)	1497 (40.4)	3314 (14.9)
Age at death, mean (SD), y	66.1 (16.7)	59.5 (14.9)	59.8 (14.9)	60.1 (14.9)	60.0 (15.1)	60.3 (15.0)	48.7 (13.4)	62.0 (13.9)	65.8 (12.4)	59.0 (14.7)

Abbreviations: ED, emergency department; LAIAs, long-acting injectable antipsychotics; NA, not applicable; OAs, oral antipsychotics.

^a^ Individuals prescribed both OAs and LAIAs (n = 23 719) experienced at least 1 of the primary and/or secondary outcome events during the observation period.

### Primary Outcomes

The direct comparison between LAIAs vs OAs is shown in Table 2. After adjustment, compared with OAs, LAIAs were associated with a significant 37% reduction in hospitalizations for any cause (IRR, 0.63 [95% CI, 0.61-0.65]), a 48% reduction in hospitalizations for psychiatric disorders (IRR, 0.52 [95% CI, 0.50-0.53]), and a 47% reduction in hospitalizations for schizophrenia (IRR, 0.53 [95% CI, 0.51-0.55]), as well as a 44% reduction in incident suicide attempts (IRR, 0.56 [95% CI, 0.44-0.71]) during the full treatment period.

**Table 2.** Results of Self-controlled Case Series Analysis for the Use of LAIAs Alone vs OAs Alone and the Risk of Outcome Events

Outcome event	Full treatment period						Subsequent treatment period^a^					
	Events, No.	Person-years	Incidence per 100 person-years	IRR (95% CI)		*P* value	Events, No.	Person-years	Incidence per 100 person-years	IRR (95% CI)		*P* value
				Unadjusted	Adjusted^b^					Unadjusted	Adjusted^b^	
Primary outcomes												
All-cause ED visits												
OAs alone	127 994	104 099	123.0	1 [Reference]	1 [Reference]	NA	102 438	91 355	112.1	1 [Reference]	1 [Reference]	NA
LAIAs alone	37 564	29 971	125.3	0.97 (0.95-0.98)	0.99 (0.97-1.00)	.09	26 613	24 356	109.3	0.96 (0.94-0.98)	0.98 (0.96-1.00)	.06
All-cause hospitalizations												
OAs alone	65 377	100 735	64.9	1 [Reference]	1 [Reference]	NA	50 334	88 470	56.9	1 [Reference]	1 [Reference]	NA
LAIAs alone	12 592	28 287	44.5	0.60 (0.59-0.62)	0.63 (0.61-0.65)	<.001	8459	22 913	36.9	0.61 (0.59-0.63)	0.64 (0.62-0.66)	<.001
Hospitalizations for psychiatric disorders												
OAs alone	40 010	94 317	42.4	1 [Reference]	1 [Reference]	NA	28 669	82 801	34.6	1 [Reference]	1 [Reference]	NA
LAIAs alone	6373	25 680	24.8	0.50 (0.48-0.52)	0.52 (0.50-0.53)	<.001	3783	20 705	18.3	0.49 (0.47-0.52)	0.50 (0.48-0.52)	<.001
Hospitalizations for schizophrenia												
OAs alone	33 436	89 539	37.3	1 [Reference]	1 [Reference]	NA	24 294	78 694	30.9	1 [Reference]	1 [Reference]	NA
LAIAs alone	5499	24 372	22.6	0.51 (0.50-0.53)	0.53 (0.51-0.55)	<.001	3303	19 644	16.8	0.50 (0.48-0.53)	0.51 (0.49-0.54)	<.001
Incident suicide attempt												
OAs alone	738	8706	8.5	1 [Reference]	1 [Reference]	NA	454	7111	6.4	1 [Reference]	1 [Reference]	NA
LAIAs alone	121	2165	5.6	0.50 (0.39-0.63)	0.56 (0.44-0.71)	<.001	70	1546	4.5	0.57 (0.42-0.78)	0.63 (0.47-0.85)	.003
Secondary outcomes												
Hospitalizations for somatic disorders												
OAs alone	34 526	71 395	48.4	1 [Reference]	1 [Reference]	NA	29 232	62 899	46.5	1 [Reference]	1 [Reference]	NA
LAIAs alone	8189	19 745	41.5	0.77 (0.75-0.80)	0.88 (0.85-0.91)	<.001	6078	15 987	38.0	0.74 (0.72-0.77)	0.87 (0.84-0.91)	<.001
Hospitalizations for cardiovascular diseases												
OAs alone	5746	21 830	26.3	1 [Reference]	1 [Reference]	NA	4946	19 516	25.3	1 [Reference]	1 [Reference]	NA
LAIAs alone	1392	6171	22.6	0.71 (0.66-0.78)	0.88 (0.81-0.96)	.006	1030	5093	20.2	0.66 (0.60-0.72)	0.83 (0.75-0.92)	<.001
Extrapyramidal symptoms												
OAs alone	15 889	112 797	14.1	1 [Reference]	1 [Reference]	NA	4365	96 937	4.5	1 [Reference]	1 [Reference]	NA
LAIAs alone	4042	32 482	12.4	0.64 (0.61-0.67)	0.86 (0.82-0.91)	<.001	483	25 964	1.9	0.34 (0.31-0.38)	0.40 (0.36-0.44)	<.001

Abbreviations: ED, emergency department; IRR, incidence rate ratio; LAIAs, long-acting injectable antipsychotics; NA, not applicable; OAs, oral antipsychotics.

^a^ Beyond the first 90 days in each treatment period.

^b^ Adjusted for age (1-year bands) and season (cut by the first date of March, May, September, and November).

Similarly, after excluding the first 90 days of each treatment period, the risk was still lower during the subsequent treatment period of LAIAs compared with OAs, suggesting that LAIAs were associated with a lower risk of hospitalizations and disease relapses than OAs and that this lower risk endured after excluding the first 90 days. However, we observed no difference in ED visits between the period in which patients were treated with LAIAs and the period in which patients were treated with OAs in either comparison (Table 2).

### Secondary Outcomes

After adjustment, compared with full treatment with OAs, full treatment with LAIAs was associated with a significant 12% reduction in hospitalizations for somatic disorders (IRR, 0.88 [95% CI, 0.85-0.91]), a 12% reduction in hospitalizations for cardiovascular diseases (IRR, 0.88 [95% CI, 0.81-0.96]), and a 14% reduction in EPSs (IRR, 0.86 [95% CI, 0.82-0.91]), suggesting that LAIAs were not associated with a higher risk of those adverse events than OAs (Table 2). During the subsequent treatment period, the results were almost the same.

### Subgroup Analyses

**Figure 2.**
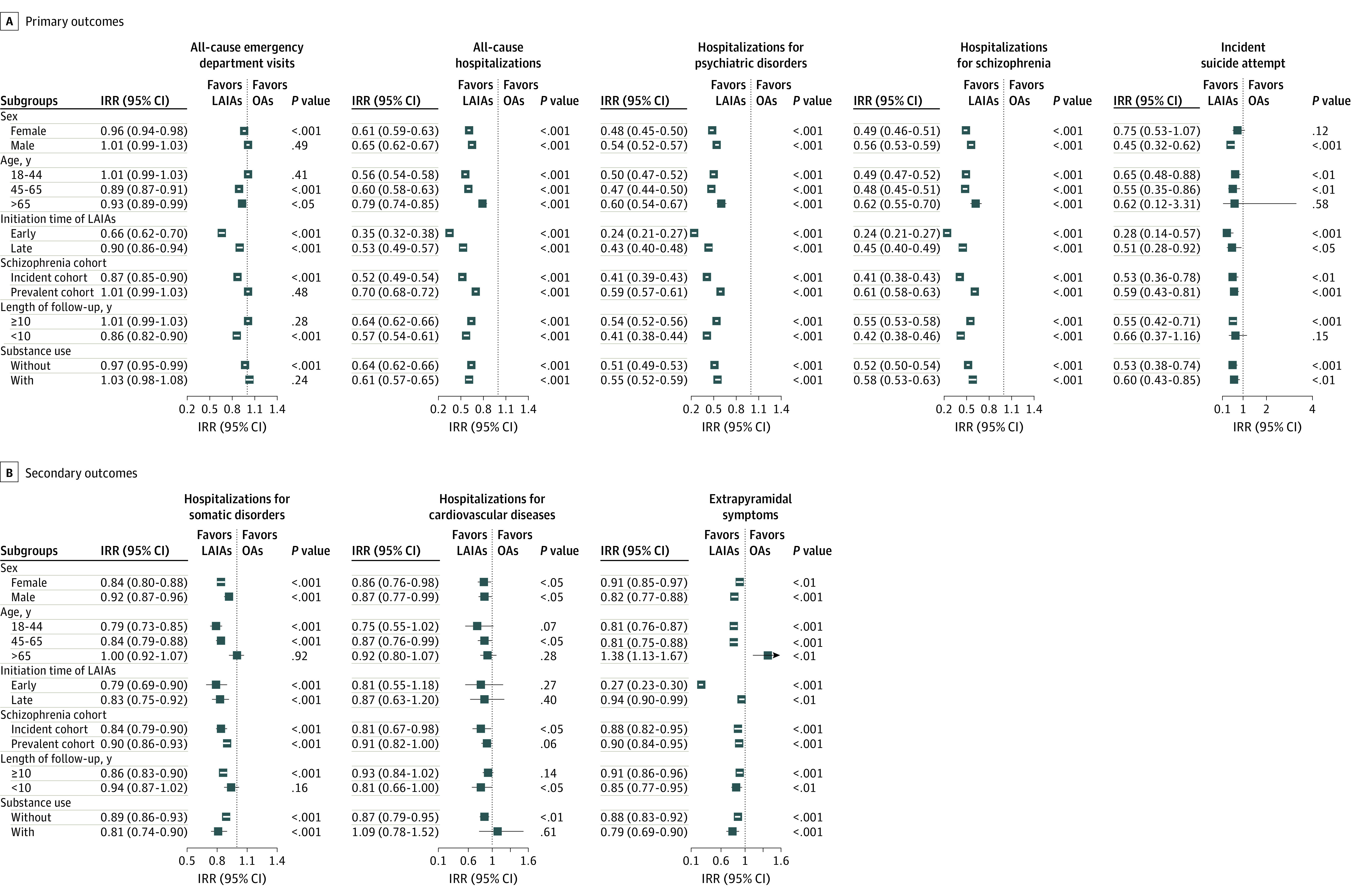
Subgroup Analyses for the Use of Long-Acting Injectable Antipsychotics (LAIAs) vs Oral Antipsychotics (OAs) During the Full Treatment Period The incidence rate ratio (IRR) estimation was adjusted for age (1-year bands) and season (cut by the first date of March, May, September, and November).

In general, the results from subgroup analyses were consistent with the main analyses (Figure 2). A similar association was observed in analyses stratified for sex. After stratifying patients by the initiation time of LAIAs, we found that early initiators had 76% fewer hospitalizations for schizophrenia during LAIA treatment than OA treatment (IRR, 0.24 [95% CI, 0.21-0.27]), while late initiators had 55% fewer hospitalizations for schizophrenia (IRR, 0.45 [95% CI, 0.40-0.49]), suggesting that early LAIA initiators could have a greater reduction in disease relapse. A greater reduction in other outcome events during the full treatment period was also observed among early initiators. People with comorbid substance use had a significantly lower risk of hospitalizations for any cause (IRR, 0.61 [95% CI, 0.57-0.65]), hospitalizations for psychiatric disorders (IRR, 0.55 [95% CI, 0.52-0.59]), hospitalizations for schizophrenia (IRR, 0.58 [95% CI, 0.53-0.63]), hospitalizations for somatic disorders (IRR, 0.81 [95% CI, 0.74-0.90]), incident suicide attempt (IRR, 0.60 [95% CI, 0.43-0.85]), and EPS (IRR, 0.79 [95% CI, 0.69-0.90]) during the period in which patients were treated with LAIAs compared with the period in which patients were treated with OAs. No significant differences in ED visits (IRR, 1.03 [95% CI, 0.98-1.08]) and hospitalizations for cardiovascular diseases (IRR, 1.09 [95% CI, 0.78-1.52]) were observed among people with comorbid substance use. Among older people (> 65 years), LAIAs were significantly associated with a lower risk of ED visits (IRR, 0.93 [95% CI, 0.89-0.99]), and hospitalizations for any cause (IRR, 0.79 [95% CI, 0.74-0.85]), hospitalizations for psychiatric disorders (IRR, 0.60 [95% CI, 0.54-0.67]), and hospitalizations for schizophrenia (IRR, 0.62 [95% CI, 0.55-0.70]), without an increased risk of hospitalizations for somatic disorders (IRR, 1.00 [95% CI, 0.92-1.07]) or hospitalizations for cardiovascular diseases (IRR, 0.92 [95% CI, 0.80-1.07]). Long-acting injectable antipsychotics were associated with a higher risk of EPS among older people (IRR, 1.38 [95% CI, 1.13-1.67]). However, after excluding the first 90 days, there was no increased risk of EPS associated with LAIAs (IRR, 0.72 [95% CI, 0.52-0.98]) (eFigure 3 in the Supplement). Patient characteristics of each subgroup are presented in eTables 3 to 9 in the Supplement.

### Additional Analyses

Generally, the results of sensitivity analyses were consistent with the main analyses (Table 3; eTable 10 in the Supplement). For instance, when people with at least 1 prescription of clozapine before the end of the observation period were removed, LAIAs were associated with lower risk of ED visits (IRR, 0.95 [95% CI, 0.93-0.96]), hospitalizations for any cause (IRR, 0.58 [95% CI, 0.57-0.60]), incident suicide attempt (IRR, 0.47 [95% CI, 0.36-0.61]), and EPS (IRR, 0.88 [95% CI, 0.84-0.93]). In the negative control analysis, no significant difference was found between LAIAs and OAs for upper respiratory tract infection (full treatment period: IRR, 0.96 [95% CI, 0.91-1.01]; subsequent treatment period: IRR, 0.97 [95% CI, 0.91-1.03]), indicating that our statistical methods were robust (Table 3). E-value analysis indicated that the results were unlikely to be associated with unmeasured confounding factors (eTable 11 in the Supplement).

**Table 3.** Results of Sensitivity Analyses and Negative Control Analysis for the Use of LAIAs Alone vs OAs Alone

Outcome event	Full treatment period		Subsequent treatment period^a^	
	Adjusted^b^ IRR (95% CI)	*P* value	Adjusted^b^ IRR (95% CI)	*P* value
**Removed people with ≥1 prescription of clozapine before end of observation period**				
All-cause ED visits	0.95 (0.93-0.96)	<.001	0.95 (0.93-0.97)	<.001
All-cause hospitalizations	0.58 (0.57-0.60)	<.001	0.60 (0.58-0.62)	<.001
Hospitalizations for psychiatric disorders	0.45 (0.43-0.46)	<.001	0.45 (0.43-0.47)	<.001
Hospitalizations for schizophrenia	0.45 (0.43-0.47)	<.001	0.45 (0.43-0.48)	<.001
Incident suicide attempt	0.47 (0.36-0.61)	<.001	0.52 (0.37-0.72)	<.001
Hospitalizations for somatic disorders	0.89 (0.85-0.92)	<.001	0.88 (0.85-0.92)	<.001
Hospitalizations for cardiovascular diseases	0.89 (0.81-0.97)	.01	0.84 (0.76-0.93)	<.001
Extrapyramidal symptoms	0.88 (0.84-0.93)	<.001	0.46 (0.42-0.52)	<.001
**Removed people with a diagnosis of bipolar disorder during observation period**				
All-cause ED visits	0.98 (0.97-1.00)	.04	0.97 (0.96-0.99)	.008
All-cause hospitalizations	0.63 (0.61-0.65)	<.001	0.64 (0.62-0.66)	<.001
Hospitalizations for psychiatric disorders	0.52 (0.50-0.53)	<.001	0.50 (0.48-0.52)	<.001
Hospitalizations for schizophrenia	0.52 (0.50-0.54)	<.001	0.51 (0.48-0.53)	<.001
Incident suicide attempt	0.53 (0.41-0.68)	<.001	0.58 (0.42-0.80)	<.001
Hospitalizations for somatic disorders	0.87 (0.84-0.91)	<.001	0.87 (0.83-0.90)	<.001
Hospitalizations for cardiovascular diseases	0.88 (0.80-0.97)	.008	0.82 (0.74-0.91)	<.001
Extrapyramidal symptoms	0.87 (0.83-0.92)	<.001	0.41 (0.37-0.45)	<.001
**Removed people who died during observation period**				
All-cause ED visits	1.00 (0.98-1.01)	.67	0.99 (0.97-1.01)	.39
All-cause hospitalizations	0.62 (0.60-0.63)	<.001	0.63 (0.61-0.65)	<.001
Hospitalizations for psychiatric disorders	0.50 (0.48-0.52)	<.001	0.48 (0.46-0.51)	<.001
Hospitalizations for schizophrenia	0.51 (0.49-0.53)	<.001	0.49 (0.47-0.52)	<.001
Incident suicide attempt	0.53 (0.41-0.69)	<.001	0.59 (0.43-0.82)	.002
Hospitalizations for somatic disorders	0.92 (0.89-0.96)	<.001	0.92 (0.88-0.97)	.001
Hospitalizations for cardiovascular diseases	1.00 (0.89-1.12)	.95	0.95 (0.83-1.08)	.42
Extrapyramidal symptoms	0.89 (0.85-0.94)	<.001	0.40 (0.36-0.45)	<.001
**First event during observation period**				
All-cause ED visits	0.62 (0.58-0.67)	<.001	0.66 (0.61-0.72)	<.001
All-cause hospitalizations	0.38 (0.36-0.41)	<.001	0.46 (0.42-0.51)	<.001
Hospitalizations for psychiatric disorders	0.30 (0.27-0.32)	<.001	0.35 (0.32-0.39)	<.001
Hospitalizations for schizophrenia	0.29 (0.27-0.32)	<.001	0.34 (0.31-0.38)	<.001
Hospitalizations for somatic disorders	1.04 (0.96-1.12)	.33	1.08 (0.99-1.19)	.07
Hospitalizations for cardiovascular diseases	1.07 (0.93-1.24)	.35	1.08 (0.92-1.28)	.32
Extrapyramidal symptoms	0.64 (0.60-0.70)	<.001	0.35 (0.29-0.42)	<.001
**Identified the cause-specific hospitalization based on principal diagnosis**				
Hospitalizations for psychiatric disorders	0.45 (0.43-0.47)	<.001	0.42 (0.40-0.44)	<.001
Hospitalizations for schizophrenia	0.45 (0.43-0.47)	<.001	0.41 (0.39-0.43)	<.001
Hospitalizations for somatic disorders	0.91 (0.88-0.95)	<.001	0.90 (0.86-0.94)	<.001
Hospitalizations for cardiovascular diseases	1.03 (0.90-1.18)	.68	1.02 (0.88-1.18)	.82
**Excluded the last 30 d before switching antipsychotic formulation**				
All-cause ED visits	0.94 (0.93-0.96)	<.001	0.96 (0.94-0.98)	<.001
All-cause hospitalizations	0.54 (0.53-0.56)	<.001	0.55 (0.53-0.56)	<.001
Hospitalizations for psychiatric disorders	0.36 (0.35-0.38)	<.001	0.35 (0.33-0.37)	<.001
Hospitalizations for schizophrenia	0.37 (0.36-0.39)	<.001	0.36 (0.34-0.38)	<.001
Incident suicide attempt	0.40 (0.29-0.54)	<.001	0.43 (0.30-0.62)	<.001
Hospitalizations for somatic disorders	0.81 (0.78-0.85)	<.001	0.80 (0.77-0.83)	<.001
Hospitalizations for cardiovascular diseases	0.81 (0.73-0.89)	<.001	0.76 (0.68-0.85)	<.001
Extrapyramidal symptoms	1.04 (0.99-1.10)	.14	0.42 (0.38-0.47)	<.001
**Added an extra 30-d washout period to each antipsychotic prescription**				
All-cause ED visits	0.97 (0.96-0.99)	<.001	0.97 (0.95-0.99)	<.001
All-cause hospitalizations	0.62 (0.60-0.64)	<.001	0.64 (0.62-0.66)	<.001
Hospitalizations for psychiatric disorders	0.49 (0.48-0.51)	<.001	0.49 (0.47-0.51)	<.001
Hospitalizations for schizophrenia	0.50 (0.48-0.52)	<.001	0.50 (0.48-0.52)	<.001
Incident suicide attempt	0.59 (0.46-0.75)	<.001	0.63 (0.47-0.84)	.002
Hospitalizations for somatic disorders	0.90 (0.86-0.93)	<.001	0.89 (0.86-0.93)	<.001
Hospitalizations for cardiovascular diseases	0.89 (0.81-0.97)	.01	0.87 (0.79-0.96)	.007
Extrapyramidal symptoms	0.76 (0.72-0.80)	<.001	0.38 (0.35-0.42)	<.001
**Among patients who switched from LAIAs to OAs at least once during observation period**				
All-cause ED visits	0.98 (0.96-1.01)	.12	1.01 (0.99-1.04)	.40
All-cause hospitalizations	0.68 (0.66-0.70)	<.001	0.69 (0.67-0.72)	<.001
Hospitalizations for psychiatric disorders	0.60 (0.58-0.62)	<.001	0.59 (0.56-0.61)	<.001
Hospitalizations for schizophrenia	0.61 (0.59-0.64)	<.001	0.60 (0.57-0.64)	<.001
Incident suicide attempt	0.60 (0.46-0.78)	<.001	0.70 (0.50-0.98)	.04
Hospitalizations for somatic disorders	0.88 (0.84-0.92)	<.001	0.89 (0.85-0.94)	<.001
Hospitalizations for cardiovascular diseases	0.84 (0.76-0.93)	<.001	0.82 (0.73-0.92)	<.001
Extrapyramidal symptoms	0.87 (0.82-0.92)	<.001	0.37 (0.33-0.42)	<.001
**Among patients who had ≥10 prescriptions of antipsychotics per year for >10 y**				
All-cause ED visits	1.01 (0.99-1.03)	.53	1.02 (0.99-1.05)	.15
All-cause hospitalizations	0.63 (0.61-0.66)	<.001	0.61 (0.58-0.64)	<.001
Hospitalizations for psychiatric disorders	0.63 (0.60-0.66)	<.001	0.60 (0.56-0.64)	<.001
Hospitalizations for schizophrenia	0.65 (0.62-0.68)	<.001	0.61 (0.57-0.65)	<.001
Incident suicide attempt	0.71 (0.50-0.99)	.04	0.77 (0.50-1.17)	.21
Hospitalizations for somatic disorders	0.73 (0.69-0.78)	<.001	0.71 (0.66-0.76)	<.001
Hospitalizations for cardiovascular diseases	0.78 (0.66-0.92)	.004	0.76 (0.63-0.92)	.005
Extrapyramidal symptoms	0.83 (0.76-0.91)	<.001	0.45 (0.38-0.54)	<.001
**Negative control analysis**				
Upper respiratory tract infection (n = 7443)	0.96 (0.91-1.01)	.12	0.97 (0.91-1.03)	.35

Abbreviations: ED, emergency department; IRR, incidence rate ratio; LAIAs, long-acting injectable antipsychotics; OAs, oral antipsychotics.

^a^ Beyond the first 90 days in each treatment period.

^b^ Adjusted for age (1-year bands) and season (cut by the first date of March, May, September, and November).

The results during the first 90 days of LAIA treatment vs the first 90 days of OA treatment were consistent with the full treatment periods and the subsequent treatment periods (eTable 12 in the Supplement). The results from the indirect comparison showed that the risk of outcome events (except EPS) during any antipsychotic treatment was higher than that during the baseline period, but lower than that during the preexposure period, and the risk was even lower during subsequent treatment periods. Compared with the preexposure period, the risk of EPS was higher during the first 90 days of antipsychotic treatment but lower during the subsequent treatment periods (eTables 13-15 in the Supplement).

## Discussion

In this large population-based SCCS study of people with schizophrenia in Hong Kong, treatment with LAIAs was associated with 44% fewer suicide attempts and 37% fewer all-cause hospitalizations during the full treatment period compared with OAs, with 48% fewer hospitalizations for psychiatric disorders and and 47% fewer hospitalizations for schizophrenia. Treatment with LAIAs was also associated with a 12% reduction in hospitalizations for somatic disorders, 12% reduction in hospitalizations for cardiovascular diseases, and 14% reduction in EPS. No significant difference was found in ED visits. Similar associations were observed during the subsequent treatment period. In the subgroup analyses, LAIAs were associated with fewer disease relapses, hospitalizations, and adverse events among older people and people with substance use, except that the risk of EPS was higher during the initial LAIA treatment (first 90 days) among older people. We also observed that early LAIAs initiators could have a greater reduction in outcome events than late initiators.

To our knowledge, this is the first study to compare the long-term risk of disease relapse, health care use, and adverse events associated with LAIAs vs OAs in an Asian population. Our findings are consistent with a large-scale Swedish study (N = 29 832) that reported 22% fewer hospitalizations with LAIAs than OAs.^11^ Our study adds further insights, as we investigated hospitalizations for different causes and safety outcomes, with findings that LAIAs were associated with not only fewer disease relapses and less health care use, but also fewer adverse events.

To our knowledge, this is also the first investigation of LAIAs during subsequent treatment periods. Bertolini et al^22^ recently reported that 40% of LAIA users discontinued LAIAs within 1 year of initiation. Of these, 33% refused treatment continuation owing to adverse events and 20% discontinued treatment owing to the clinician’s decision that LAIAs were no longer required. Therefore, data on the subsequent use of LAIAs and their safety profiles are important for guidance on whether patients should continue LAIAs. In addition, because LAIAs are not typically used as first-line treatment, baseline disease severity is usually different when initiating LAIAs vs initiating OAs, and the initial treatment period should be excluded to reduce potential bias; this has not been taken into account in previous studies. After excluding the first 90 days of each treatment, the absolute risk of outcome events was much lower for both OAs and LAIAs, but the relative risk (LAIAs vs OAs) was still less than 1 (Table 2). This finding shows that the lower risk of hospitalization and disease relapse and better or comparable safety outcomes associated with LAIAs (compared with OAs) is sustained after the first 90 days, supporting the long-term use of LAIAs in subsequent treatment.

Few studies have investigated the use of LAIAs in special patient groups, to our knowledge. Our study demonstrated the advantages associated with LAIAs vs OAs among people with substance use, a group with a high risk of nonadherence and repeated relapse.^24,25^ We also explored the use of LAIAs vs OAs in older people, who are a vulnerable group often facing particular difficulties with drug interactions, altered pharmacokinetics, and medication adverse effects.^23^ Lin et al^37^ compared LAIAs and OAs on time to rehospitalization within 1 year of hospital discharge among older people from a single hospital. Our study demonstrated a lower risk of hospitalizations and disease relapse associated with LAIAs among older people over a long follow-up, and further found that LAIAs were not associated with an increased risk of hospitalizations for somatic disorders and hospitalizations for cardiovascular diseases. However, caution should be exercised among older people when newly commencing LAIAs, given the increased risk of EPS during the first 90 days of treatment with LAIAs. Finally, our data suggested that early LAIA initiators might have greater reductions than late LAIAs initiators in outcome events, consistent with emerging data from randomized clinical trials and schizophrenia treatment guidelines.^8,38^

### Strengths and Limitations

Our study has some strengths. It accounted for several methodological issues in previous studies from Asia. We adopted a within-individual comparison because people with schizophrenia often switched antipsychotic medications, and traditional cohort studies using between-individual comparison could not overcome this misclassification bias.^12,14,15,16,17,21^ Selection bias is another problem in between-individual comparison because substantial differences exist in the inherent characteristics (eg, adherence) of patients prescribed LAIAs and patients prescribed OAs.^12,14,15,16,17,21^ Within-individual comparisons could overcome this problem and only time-varying confounders need to be adjusted. Although 1 Korean study^18^ has applied a within-individual comparison, no adjustments for the time-varying confounders were made, and misclassification bias also existed because people receiving combined LAIA and OA therapy were categorized as exposed to LAIAs and compared with OA monotherapy. Our SCCS study compared the periods when individuals were exposed to either LAIAs alone vs OAs alone. We further adjusted for time-varying confounders (age and season) and assessed the potential associations of other unmeasured confounding associated with the outcomes using the E-value.

There are also several limitations to our study. First, we assessed only the pooled estimates for all LAIAs rather than for individual antipsychotics, and although we ran the analyses including and excluding clozapine, the results were similar. Future studies should compare specific LAIAs. Second, the dose of antipsychotics was not accounted for because the dosage information was recorded in a different way for LAIAs and OAs (eg, LAIA dosage was recorded as a total dose over a time period, while OA dosage was recorded as a daily divided dose). A robust method to calculate the dose of LAIAs should be developed for future studies. Third, although we adjusted for age and season, other residual time-varying confounders could still be unaccounted for (eg, comorbidities, contaminant medications). However, most time-varying confounders were considered not correlated with the use of different formulations of antipsychotics and outcome events. E-values also indicated that the results were unlikely to be associated with unmeasured confounding factors. Fourth, some patients may have sought health care in private hospitals, potentially leading to loss to follow-up. However, the Hospital Authority is the major health care provider in Hong Kong and covers more than 80% of all hospital admissions.^27^ The potential for missing data because of loss to follow-up was considered unlikely to be associated with our conclusions generated from a large data set. Fifth, only people from Hong Kong were included, which was not representative of the whole Asian population. A recent study found a wide variation in the prevalence of LAIAs between Asian regions.^6^ It is worth investigating the medication preference and clinical outcomes of people treated with LAIAs by performing multinational studies.

## Conclusions

In this SCCS study of people in Hong Kong with schizophrenia, our findings reinforced that LAIAs were associated with a lower risk of hospitalizations, disease relapses, and suicide attempts than OAs, and this association remained during subsequent treatment periods. No significant difference was found in ED visits. Long-acting injectable antipsychotics had comparable or better safety profiles than OAs. These results were robust in sub-analyses of older people and people with substance use, except for a higher risk of EPS during the initial treatment of LAIAs in older people, among whom caution should be exercised when initiating LAIAs. Our findings supported expanding the long-term use of LAIAs in the Chinese population with schizophrenia.

## Notes

Supplement. **eMethods.** Methods for the Subgroup Analyses, Sensitivity Analyses, Negative Control Analysis and E-Value Calculation **eTable 1.** Half-lives of the Antipsychotics Used in Hong Kong Public Hospitals **eTable 2.** Definition of the Outcome Events **eTable 3.** Patient Characteristics by Sex **eTable 4.** Patient Characteristics by Age, for the Primary Outcomes **eTable 5.** Patient Characteristics by Age, for the Secondary Outcomes **eTable 6.** Patient Characteristics by Initiation Time of LAIAs **eTable 7.** Patient Characteristics by Schizophrenia Cohort **eTable 8.** Patient Characteristics by Length of Follow-up **eTable 9.** Patient Characteristics by Substance Use **eTable 10.** Sensitivity Analysis by Restricting the Age Range of Early/Late LAIAs Initiators **eTable 11.** Results From E-Value Analyses **eTable 12.** Results of Self-controlled Case Series Analysis for the Use of LAIAs Alone vs OAs Alone and the Risk of Outcome Events, During the First 90 Days of Treatment **eTable 13.** Results of Self-controlled Case Series Analysis for the Full Treatment Period of OAs Alone, Full Treatment Period of LAIAs Alone, and Full Treatment Period of the Combination Use of OAs Plus LAIAs vs Baseline Period or Preexposure Period, and the Risk of Outcome Events **eTable 14.** Results of Self-controlled Case Series Analysis for the First 90 Days of OAs Alone, the Subsequent Use of OAs Alone, the First 90 Days of LAIAs Alone, the Subsequent Use of LAIAs Alone, the First 90 Days of OAs Plus LAIAs, the Subsequent Use of OAs Plus LAIAs vs Baseline Period or Preexposure Period, and the Risk of Outcome Events **eTable 15.** The Rate of Outcome Events During Different Risk Windows **eFigure 1.** Illustration of Self-controlled Case Series Study for Indirect Comparison **eFigure 2.** Patient Identification **eFigure 3.** Subgroup Analyses for the Use of LAIAs vs OAs and Risk of Outcomes, During the Subsequent Treatment Period
eReferences.
