# Supplementary material for: Association of Long-Acting Injectable Antipsychotics and Oral Antipsychotics With Disease Relapse, Health Care Use, and Adverse Events Among People With Schizophrenia
Source: JAMA Netw Open. 2022 Jul 28;5(7):e2224163. doi: 10.1001/jamanetworkopen.2022.24163 (PMC9335136; doi:10.1001/jamanetworkopen.2022.24163)
Supplement: Supplement. — eMethods. Methods for the Subgroup Analyses, Sensitivity Analyses, Negative Control Analysis and E-Value Calculation eTable 1. Half-lives of the Antipsychotics Used in Hong Kong Public Hospitals eTable 2. Definition of the Outcome Events eTable 3. Patient Characteristics by Sex eTable 4. Patient Characteristics by Age, for the Primary Outcomes eTable 5. Patient Characteristics by Age, for the Secondary Outcomes eTable 6. Patient Characteristics by Initiation Time of LAIAs eTable 7. Patient Characteristics by Schizophrenia Cohort eTable 8. Patient Characteristics by Length of Follow-up eTable 9. Patient Characteristics by Substance Use eTable 10. Sensitivity Analysis by Restricting the Age Range of Early/Late LAIAs Initiators eTable 11. Results From E-Value Analyses eTable 12. Results of Self-controlled Case Series Analysis for the Use of LAIAs Alone vs OAs Alone and the Risk of Outcome Events, During the First 90 Days of Treatment eTable 13. Results of Self-controlled Case Series Analysis for the Full Treatment Period of OAs Alone, Full Treatment Period of LAIAs Alone, and Full Treatment Period of the Combination Use of OAs Plus LAIAs vs Baseline Period or Preexposure Period, and the Risk of Outcome Events eTable 14. Results of Self-controlled Case Series Analysis for the First 90 Days of OAs Alone, the Subsequent Use of OAs Alone, the First 90 Days of LAIAs Alone, the Subsequent Use of LAIAs Alone, the First 90 Days of OAs Plus LAIAs, the Subsequent Use of OAs Plus LAIAs vs Baseline Period or Preexposure Period, and the Risk of Outcome Events eTable 15. The Rate of Outcome Events During Different Risk Windows eFigure 1. Illustration of Self-controlled Case Series Study for Indirect Comparison eFigure 2. Patient Identification eFigure 3. Subgroup Analyses for the Use of LAIAs vs OAs and Risk of Outcomes, During the Subsequent Treatment Period eReferences. [file jamanetwopen-e2224163-s001.pdf]

Supplementary Online Content

Wei Y, Yan VKC, Kang W, et al. Association of long-acting injectable antipsychotics and oral antipsychotics with disease relapse, health care use, and adverse events among people with schizophrenia. *JAMA Netw Open.* 2022;5(7):e2224163.
doi:10.1001/jamanetworkopen.2022.24163

eMethods. Methods for the Subgroup Analyses, Sensitivity Analyses, Negative Control Analysis and E-Value Calculation
eTable 1. Half-lives of the Antipsychotics Used in Hong Kong Public Hospitals
eTable 2. Definition of the Outcome Events
eTable 3. Patient Characteristics by Sex
eTable 4. Patient Characteristics by Age, for the Primary Outcomes
eTable 5. Patient Characteristics by Age, for the Secondary Outcomes
eTable 6. Patient Characteristics by Initiation Time of LAIAs
eTable 7. Patient Characteristics by Schizophrenia Cohort
eTable 8. Patient Characteristics by Length of Follow-up
eTable 9. Patient Characteristics by Substance Use
eTable 10. Sensitivity Analysis by Restricting the Age Range of Early/Late LAIAs Initiators
eTable 11. Results From E-Value Analyses
eTable 12. Results of Self-controlled Case Series Analysis for the Use of LAIAs Alone vs OAs Alone and the Risk of Outcome Events, During the First 90 Days of Treatment
eTable 13. Results of Self-controlled Case Series Analysis for the Full Treatment Period of OAs Alone, Full Treatment Period of LAIAs Alone, and Full Treatment Period of the Combination Use of OAs plus LAIAs vs Baseline Period or Preexposure Period, and the Risk of Outcome Events
eTable 14. Results of Self-controlled Case Series Analysis for the First 90 Days of OAs Alone, the Subsequent Use of OAs Alone, the First 90 Days of LAIAs Alone, the Subsequent Use of LAIAs Alone, the First 90 Days of OAs Plus LAIAs, the Subsequent Use of OAs Plus LAIAs vs Baseline Period or Preexposure Period, and the Risk of Outcome Events
eTable 15. The Rate of Outcome Events During Different Risk Windows
eFigure 1. Illustration of Self-controlled Case Series Study for Indirect Comparison
eFigure 2. Patient Identification
eFigure 3. Subgroup Analyses for the Use of LAIAs vs OAs and Risk of Outcomes, During the Subsequent Treatment Period
eReferences.

This supplementary material has been provided by the authors to give readers additional information about their work.

© 2022 Wei Y et al. *JAMA Network Open.*

eMethods. Methods for the Subgroup Analyses, Sensitivity Analyses, Negative Control Analysis and E-Value Calculation

Subgroup analyses

1. We performed a subgroup analysis by sex to evaluate the associations in males and females.
2. For the subgroup analysis by age, the individual observation period was trimmed based on age. The follow-up for the group of 18-44 years began on January 1, 2004, date of the first diagnosis of schizophrenia or 18th birthday (whichever came later) and ended on December 31, 2019, date of death or 45th birthday (whichever came first). The follow-up for the group of 45-65 years began on January 1, 2004, date of the first diagnosis of schizophrenia or 45th birthday (whichever came later) and ended on December 31, 2019, date of death or 65th birthday (whichever came first). The follow-up for the group of over 65 years began on January 1, 2004, date of the first diagnosis of schizophrenia or 65th birthday (whichever came later) and ended on December 31, 2019 or date of death (whichever came first).
3. Another subgroup analysis was conducted according to the time since the first diagnosis of schizophrenia to the initiation of LAIAs. The early LAIAs initiators were defined as people who started a LAIA within two years of the first diagnosis of schizophrenia, while the late LAIAs initiators were defined as those starting LAIAs after two years. Since the prescription information before 2002 in CDARS is not well-recorded and the date of the first schizophrenia diagnosis in the prevalent cohort (diagnosed with schizophrenia before January 1, 2004) may not be accurate, only people in the incident cohort (newly

© 2022 Wei Y et al. *JAMA Network Open.*

diagnosed with schizophrenia on or after January 1, 2004) were included in this subgroup analysis.

4. According to the time of the first diagnosis of schizophrenia, we divided the cohort into prevalent cohort (date of first schizophrenia diagnosis before January 1, 2004 [not including this date]) and incident cohort (date of first schizophrenia diagnosis on or after January 1, 2004), to assess whether different associations were observed among the incident patients.
5. To avoid bias from people who were followed for a very short period, we stratified the cohort into the subgroup with long follow-up (⩾10 years) and short follow-up (<10 years). The length of follow-up was defined by the start date and end date of the individual observation period.
6. We further performed a subgroup analysis by substance use. People with substance use were defined as those diagnosed with substance use (ICD-9-CM 292, 304, 305.2-305.9) before the end of the observation period (any time before or during the observation period).

Sensitivity analyses

1. Since clozapine is mainly used to manage treatment-resistant psychosis, the disease profiles of people prescribed clozapine could be different from those treated with other antipsychotics. However, there is no long-acting formulation for clozapine, and most patients stay on clozapine long-term. Therefore, we repeated the analysis after excluding individuals with at least one prescription of clozapine before the end of the observation period.

© 2022 Wei Y et al. *JAMA Network Open.*

2. Since the diagnosis of bipolar disorder (ICD-9-CM 296.1, 296.4, 296.5, 296.6, 296.7, 296.8) and the diagnosis of schizophrenia (ICD-9-CM 295) are mutually exclusive in clinical practice, people who were diagnosed with bipolar disorder during the observation period should not be included in the analysis after the diagnosis. Therefore, we repeated the analysis after excluding individuals diagnosed with bipolar disorders during the observation period.
3. People who died during the observation period were excluded in the sensitivity analysis to further reduce the potential bias from the event-dependent observation period.
4. We repeated the analyses for the first outcome event during the observation period to control for any correlations between these events and to test for any violation of the SCCS assumption that the outcome events should be independent.
5. The secondary diagnoses of hospitalization were usually documented for the history of the disease, so we included only those hospitalizations with an eligible principal diagnosis in the sensitivity analysis.
6. Non-adherence is usually an indication of switching from OAs to LAIAs, and an adverse event may lead to a switch of antipsychotics, which could introduce event-dependent exposure and violate the SCCS assumption that the occurrence of an event should not influence the subsequent exposure. Hence, we repeated the analysis after excluding the 30-day antipsychotic treatment before switching formulations.
7. Although a washout period of five half-lives of each antipsychotic had been added to account for any residual treatment effect in the main analysis, we added an extra washout period of 30 days to further account for the residual effect.

© 2022 Wei Y et al. *JAMA Network Open.*

8. It was reported that patients usually discontinued LAIAs due to various reasons.[1] To detect any potential cohort effect among these patients, we repeated the analysis among patients who switched from LAIAs (at least 30 days) to OAs (at least 30 days) at least once during the observation period.
9. Compared with patients with only a few antipsychotic prescriptions, people who visited hospitals regularly to obtain antipsychotic prescriptions over a long period were more likely to have better adherence and the discrepancy between their prescription records and the actual medication administration was more minor. Therefore, we repeated the analysis among patients who had at least 10 prescriptions of antipsychotics per year for over 5 years.
10. In consideration that the first diagnosis of schizophrenia might be delayed in registry data, we further restricted patients in the subgroup analysis by initiation time of LAIAs (Subgroup analysis 3) to those newly diagnosed with schizophrenia at age 18-35 years.

Negative control analysis

- Additionally, we performed a negative control analysis by using upper respiratory tract infection (ICD-9-CM 010-012, 031.0, 032.1-3, 033-034, 039.1, 040.1, 460-466, 472-476, 480-488, 490, 491) as the outcome events to validate our results, since there is no evidence of an association between the formulation of antipsychotics and upper respiratory tract infections.

E-value calculation

- E-value was calculated to assess the potential effects of any unmeasured confounding, which was defined as the minimum strength of association that an unmeasured

© 2022 Wei Y et al. *JAMA Network Open.*

confounder would need to have with both treatment and outcome, conditional on the measured covariates, to explain away an observed association.[2]

© 2022 Wei Y et al. *JAMA Network Open.*

eTable 1. Half-lives of the Antipsychotics Used in Hong Kong Public Hospitals

Drug name	Elimination half-life (hours)
Amisulpride	12
Aripiprazole	94
Asenapine	24
Chlorpromazine	30
Clopenthixol	28
Clozapine	14
Flupenthixol	35
Fluphenazine	58
Haloperidol	24
Lurasidone	40
Olanzapine	33
Paliperidone	23
Pericyazine	12
Perphenazine	20
Pimozide	55
Quetiapine	12
Risperidone	20
Sertindole	72
Sulpiride	8
Thioridazine	30
Thiothixene	34
Trifluoperazine	20
Ziprasidone	10
Zuclopenthixol	28

© 2022 Wei Y et al. *JAMA Network Open.*

eTable 2. Definition of the Outcome Events

**Outcomes**	**Data source**	**Definition**
All-cause emergency department visits	All emergency department visit records	Defined as an emergency department attendance for any cause
All-cause hospitalizations	All inpatient admission records	Defined as a hospitalization for any cause
Hospitalizations for any psychiatric disorders	All inpatient admission records	Defined as a hospitalization with ICD-9-CM diagnosis code: 290-319
Hospitalizations for schizophrenia	All inpatient admission records	Defined as a hospitalization with ICD-9-CM diagnosis code: 295
Incident suicide attempt	All diagnosis	Defined as the first suicide attempt during the observation period of patients who had never had suicide attempts before the observation period. The ICD-9-CM diagnosis code for a suicide attempt is E950-E959.
Hospitalizations for any somatic disorders	All inpatient admission records	Defined as a hospitalization with ICD-9-CM diagnosis code: 000-289, 320-999
Hospitalizations for cardiovascular diseases	All inpatient admission records	Defined as a hospitalization with ICD-9-CM diagnosis code: 390-459
Extrapyramidal symptoms	All diagnosis	Defined as a new prescription of the medications for extrapyramidal symptoms (>180 days gap between the previous prescription for extrapyramidal symptoms). Medications used for extrapyramidal symptoms included trihexyphenidyl, benztropine, amantadine and biperiden.[3, 4]

Abbreviations: ICD-9-CM, International Classification of Diseases, Ninth Revision, Clinical Modification.

© 2022 Wei Y et al. *JAMA Network Open.*

eTable 3. Patient Characteristics by Sex

Patient characteristics	Individuals prescribed both OAs and LAIAs, with at least one of the following outcome events during the observation period															
	Primary outcomes										Secondary outcomes					
	**All-cause ED visits**		**All-cause hospitalizations**		**Hospitalizations for psychiatric disorders**		**Hospitalizations for schizophrenia**		**Incident suicide attempt**		**Hospitalizations for somatic disorders**		**Hospitalizations for cardiovascular diseases**		**Extrapyramidal symptoms**	
	Female	Male	Female	Male	Female	Male	Female	Male	Female	Male	Female	Male	Female	Male	Female	Male
**No. of patients**	10551	11462	10110	10863	9317	9966	8859	9526	613	840	7420	7976	1653	2057	10646	11536
**Age, mean (SD), y**																
**At first schizophrenia diagnosis**	40.10 (12.93)	37.81 (12.60)	40.10 (12.99)	37.87 (12.72)	39.71 (12.96)	37.40 (12.69)	39.74 (12.96)	37.48 (12.70)	34.34 (11.57)	32.57 (10.27)	41.55 (13.43)	39.72 (13.04)	48.65 (13.53)	46.38 (12.34)	39.90 (12.53)	37.54 (12.30)
**At cohort entry**	42.75 (12.95)	40.77 (12.82)	42.75 (13.01)	40.83 (12.94)	42.34 (13.00)	40.35 (12.92)	42.40 (13.01)	40.47 (12.93)	36.94 (11.65)	35.42 (10.64)	44.51 (13.36)	42.93 (13.12)	52.06 (13.16)	50.09 (11.88)	42.51 (12.52)	40.47 (12.51)
**At time of event**	48.53 (13.83)	47.66 (13.57)	51.44 (15.14)	50.50 (14.88)	48.35 (14.66)	47.20 (14.38)	48.79 (14.59)	48.12 (14.36)	42.21 (12.45)	40.51 (11.83)	56.11 (14.81)	55.95 (14.04)	63.81 (12.86)	61.31 (11.89)	44.19 (12.81)	41.79 (12.77)
**Death**																
**No. (%) of patients who died during the observation period**	1356 (12.85)	2156 (18.81)	1314 (13.00)	2065 (19.01)	1181 (12.68)	1822 (18.28)	1119 (12.63)	1748 (18.35)	77 (12.56)	159 (18.93)	1170 (15.77)	1844 (23.12)	585 (35.39)	912 (44.34)	1263 (11.86)	2051 (17.78)
**Age at death, mean (SD), y**	61.36 (15.47)	58.87 (14.41)	61.54 (15.47)	59.20 (14.39)	61.30 (15.72)	59.07 (14.69)	61.56 (15.62)	59.44 (14.49)	48.44 (14.54)	48.78 (12.92)	63.52 (14.60)	61.06 (13.32)	67.83 (13.38)	64.47 (11.52)	60.47 (15.24)	57.98 (14.31)

Abbreviations: OAs, oral antipsychotics; LAIAs, long-acting injectable antipsychotics; ED, emergency department; SD, standard deviation

© 2022 Wei Y et al. *JAMA Network Open.*

eTable 4. Patient Characteristics by Age, for the Primary Outcomes

Patient characteristics	Individuals prescribed both OAs and LAIAs, with at least one of the following outcome events during the observation period														
	Primary outcomes														
	All-cause ED visits			All-cause hospitalizations			Hospitalizations for psychiatric disorders			Hospitalizations for schizophrenia			Incident suicide attempt		
	18-44 years	45-65 years	> 65 years	18-44 years	45-65 years	> 65 years	18-44 years	45-65 years	> 65 years	18-44 years	45-65 years	> 65 years	18-44 years	45-65 years	> 65 years
**No. of patients**	13330	15419	4855	12660	14636	4668	11912	13264	4140	11304	12703	4004	1132	885	146
**Sex, No. (%)**															
**Female**	6110 (45.84)	7592 (49.24)	2516 (51.82)	5845 (46.17)	7244 (49.49)	2415 (51.74)	5508 (46.24)	6597 (49.74)	2142 (51.74)	5215 (46.13)	6291 (49.52)	2062 (51.50)	465 (41.08)	382 (43.16)	71 (48.63)
**Male**	7220 (54.16)	7827 (50.76)	2339 (48.18)	6815 (53.83)	7392 (50.51)	2253 (48.26)	6404 (53.76)	6667 (50.26)	1998 (48.26)	6089 (53.87)	6412 (50.48)	1942 (48.50)	667 (58.92)	503 (56.84)	75 (51.37)
**Age, mean (SD), y**															
**At first schizophrenia diagnosis**	30.79 (7.31)	41.71 (9.39)	55.51 (8.69)	30.72 (7.32)	41.75 (9.42)	55.59 (8.74)	30.55 (7.32)	41.53 (9.44)	55.53 (8.72)	30.55 (7.31)	41.53 (9.44)	55.50 (8.69)	28.98 (7.16)	38.43 (8.91)	52.75 (7.09)
**At cohort entry**	33.40 (7.45)	48.59 (5.11)	66.30 (3.37)	33.33 (7.45)	48.63 (5.14)	66.33 (3.41)	33.13 (7.46)	48.53 (5.10)	66.31 (3.34)	33.16 (7.46)	48.55 (5.11)	66.29 (3.30)	31.58 (7.31)	47.15 (4.08)	65.42 (1.67)
**At time of event**	48.06 (13.70)	48.06 (13.70)	48.06 (13.70)	50.94 (15.01)	50.94 (15.01)	50.94 (15.01)	47.73 (14.52)	47.73 (14.52)	47.73 (14.52)	48.43 (14.47)	48.43 (14.47)	48.43 (14.47)	41.23 (12.12)	41.23 (12.12)	41.23 (12.12)
**Death**															
**No. (%) of patients who died during the observation period**	1111 (8.33)	2301 (14.92)	1313 (27.04)	1054 (8.33)	2218 (15.15)	1288 (27.59)	985 (8.27)	1959 (14.77)	1148 (27.73)	920 (8.14)	1887 (14.85)	1115 (27.85)	153 (13.52)	134 (15.14)	22 (15.07)
**Age at death, mean (SD), y**	43.46 (9.43)	60.35 (7.89)	74.64 (6.99)	43.61 (9.44)	60.44 (7.92)	74.72 (7.01)	43.56 (9.55)	60.62 (8.03)	74.82 (7.04)	43.88 (9.48)	60.71 (8.04)	74.81 (6.97)	41.59 (9.47)	56.71 (7.07)	72.28 (6.61)

Abbreviations: OAs, oral antipsychotics; LAIAs, long-acting injectable antipsychotics; ED, emergency department; SD, standard deviation

© 2022 Wei Y et al. *JAMA Network Open.*

eTable 5. Patient Characteristics by Age, for the Secondary Outcomes

Patient characteristics	Individuals prescribed both OAs and LAIAs, with at least one of the following outcome events during the observation period								
	Secondary outcomes								
	Hospitalizations for somatic disorders			Hospitalizations for cardiovascular diseases			Extrapyramidal symptoms		
	18-44 years	45-65 years	> 65 years	18-44 years	45-65 years	> 65 years	18-44 years	45-65 years	> 65 years
**No. of patients**	8312	11296	4252	1157	2912	1718	13579	15664	4649
**Sex, No. (%)**									
**Female**	3851 (46.33)	5508 (48.76)	2191 (51.53)	480 (41.49)	1238 (42.51)	829 (48.25)	6213 (45.75)	7756 (49.51)	2411 (51.86)
**Male**	4461 (53.67)	5788 (51.24)	2061 (48.47)	677 (58.51)	1674 (57.49)	889 (51.75)	7366 (54.25)	7908 (50.49)	2238 (48.14)
**Age, mean (SD), y**									
**At first schizophrenia diagnosis**	30.95 (7.28)	42.40 (9.54)	55.89 (8.79)	33.27 (6.77)	45.54 (9.09)	57.38 (8.97)	30.90 (7.32)	41.68 (9.32)	54.96 (8.30)
**At cohort entry**	33.86 (7.40)	49.10 (5.37)	66.42 (3.52)	36.91 (6.62)	50.99 (5.93)	66.88 (3.94)	33.50 (7.43)	48.50 (5.02)	66.07 (3.00)
**At time of event**	56.02 (14.42)	56.02 (14.42)	56.02 (14.42)	62.39 (12.38)	62.39 (12.38)	62.39 (12.38)	42.97 (12.84)	42.97 (12.84)	42.97 (12.84)
**Death**									
**No. (%) of patients who died during the observation period**	806 (9.70)	2055 (18.19)	1266 (29.77)	289 (24.98)	1033 (35.47)	771 (44.88)	1111 (8.18)	2203 (14.06)	1152 (24.78)
**Age at death, mean (SD), y**	45.40 (8.82)	60.81 (7.89)	74.79 (7.03)	48.98 (7.81)	62.24 (7.63)	75.38 (7.06)	43.39 (9.39)	59.99 (7.92)	74.30 (6.84)

Abbreviations: OAs, oral antipsychotics; LAIAs, long-acting injectable antipsychotics; ED, emergency department; SD, standard deviation

© 2022 Wei Y et al. *JAMA Network Open.*

eTable 6. Patient Characteristics by Initiation Time of LAIAs

Patient characteristics	Individuals prescribed both OAs and LAIAs, with at least one of the following outcome events during the observation period															
	Primary outcomes										Secondary outcomes					
	**All-cause ED visits**		**All-cause hospitalizations**		**Hospitalizations for psychiatric disorders**		**Hospitalizations for schizophrenia**		**Incident suicide attempt**		**Hospitalizations for somatic disorders**		**Hospitalizations for cardiovascular diseases**		**Extrapyramidal symptoms**	
	Early initiation	Late initiation	Early initiation	Late initiation	Early initiation	Late initiation	Early initiation	Late initiation	Early initiation	Late initiation	Early initiation	Late initiation	Early initiation	Late initiation	Early initiation	Late initiation
**No. of patients**	2617	3478	2615	3407	2501	3316	2333	3135	153	308	1509	2109	253	345	2663	3348
**Sex, No. (%)**																
**Female**	1349 (51.55)	1824 (52.44)	1369 (52.35)	1796 (52.71)	1312 (52.46)	1756 (52.96)	1224 (52.46)	1664 (53.08)	68 (44.44)	137 (44.48)	787 (52.15)	1103 (52.30)	116 (45.85)	174 (50.43)	1390 (52.20)	1757 (52.48)
**Male**	1268 (48.45)	1654 (47.56)	1246 (47.65)	1611 (47.29)	1189 (47.54)	1560 (47.04)	1109 (47.54)	1471 (46.92)	85 (55.56)	171 (55.52)	722 (47.85)	1006 (47.70)	137 (54.15)	171 (49.57)	1273 (47.80)	1591 (47.52)
**Age, mean (SD), y**																
**At first schizophrenia diagnosis**	39.50 (13.64)	35.62 (12.72)	39.68 (13.59)	35.68 (12.73)	39.58 (13.58)	35.56 (12.70)	39.47 (13.57)	35.54 (12.73)	34.66 (12.57)	32.41 (10.53)	41.53 (14.43)	37.38 (13.59)	50.83 (14.22)	47.12 (14.62)	39.17 (13.07)	35.47 (12.51)
**At cohort entry**	39.50 (13.64)	35.62 (12.72)	39.68 (13.59)	35.68 (12.73)	39.58 (13.58)	35.56 (12.70)	39.47 (13.57)	35.54 (12.73)	34.66 (12.57)	32.41 (10.53)	41.53 (14.43)	37.38 (13.59)	50.83 (14.22)	47.12 (14.62)	39.17 (13.07)	35.47 (12.51)
**At time of event**	42.85 (14.78)	41.33 (13.69)	45.19 (15.79)	42.98 (15.28)	42.05 (14.80)	40.50 (14.14)	42.36 (14.85)	41.13 (14.43)	37.62 (12.78)	37.05 (11.16)	51.43 (15.86)	48.80 (16.27)	60.36 (13.65)	58.55 (14.88)	39.66 (13.11)	37.46 (12.67)
**Death**																
**No. (%) of patients who died during the observation period**	280 (10.70)	278 (7.99)	273 (10.44)	274 (8.04)	254 (10.16)	267 (8.05)	236 (10.12)	253 (8.07)	27 (17.65)	29 (9.42)	209 (13.85)	207 (9.82)	86 (33.99)	94 (27.25)	260 (9.76)	262 (7.83)
**Age at death, mean (SD), y**	53.30 (17.65)	51.48 (16.70)	53.46 (17.73)	51.73 (16.61)	53.46 (17.69)	51.63 (16.72)	53.69 (17.59)	51.55 (16.73)	45.86 (16.64)	43.07 (12.40)	57.54 (16.83)	55.40 (16.25)	62.92 (15.33)	60.85 (15.24)	51.87 (17.34)	51.00 (16.61)

Abbreviations: OAs, oral antipsychotics; LAIAs, long-acting injectable antipsychotics; ED, emergency department; SD, standard deviation

© 2022 Wei Y et al. *JAMA Network Open.*

eTable 7. Patient Characteristics by Schizophrenia Cohort

Patient characteristics	Individuals prescribed both OAs and LAIAs, with at least one of the following outcome events during the observation period															
	Primary outcomes										Secondary outcomes					
	All-cause ED visits		All-cause hospitalizations		Hospitalizations for psychiatric disorders		Hospitalizations for schizophrenia		Incident suicide attempt		Hospitalizations for somatic disorders		Hospitalizations for cardiovascular diseases		Extrapyramidal symptoms	
	Incident cohort	Prevalent cohort	Incident cohort	Prevalent cohort	Incident cohort	Prevalent cohort	Incident cohort	Prevalent cohort	Incident cohort	Prevalent cohort	Incident cohort	Prevalent cohort	Incident cohort	Prevalent cohort	Incident cohort	Prevalent cohort
**No. of patients**	8873	13140	8477	12496	7925	11358	7459	10926	576	877	5430	9966	986	2724	9099	13083
**Sex, No. (%)**																
**Female**	4562 (51.41)	5989 (45.58)	4385 (51.73)	5725 (45.81)	4112 (51.89)	5205 (45.83)	3869 (51.87)	4990 (45.67)	264 (45.83)	349 (39.79)	2784 (51.27)	4636 (46.52)	480 (48.68)	1173 (43.06)	4698 (51.63)	5948 (45.46)
**Male**	4311 (48.59)	7151 (54.42)	4092 (48.27)	6771 (54.19)	3813 (48.11)	6153 (54.17)	3590 (48.13)	5936 (54.33)	312 (54.17)	528 (60.21)	2646 (48.73)	5330 (53.48)	506 (51.32)	1551 (56.94)	4401 (48.37)	7135 (54.54)
**Age, mean (SD), y**																
**At first schizophrenia diagnosis**	39.11 (13.64)	38.77 (12.22)	39.16 (13.70)	38.80 (12.33)	38.76 (13.65)	38.35 (12.30)	38.74 (13.67)	38.46 (12.29)	33.99 (11.56)	32.88 (10.37)	41.37 (14.39)	40.18 (12.58)	50.44 (13.83)	46.28 (12.41)	39.00 (13.21)	38.45 (11.93)
**At cohort entry**	39.11 (13.64)	43.48 (12.09)	39.16 (13.70)	43.51 (12.21)	38.76 (13.65)	43.09 (12.22)	38.74 (13.67)	43.22 (12.21)	33.99 (11.56)	37.42 (10.57)	41.37 (14.39)	44.95 (12.42)	50.44 (13.83)	51.16 (11.99)	39.00 (13.21)	43.15 (11.79)
**At time of event**	43.88 (14.50)	49.81 (12.95)	46.05 (15.87)	53.29 (13.99)	42.64 (14.80)	50.44 (13.62)	43.26 (14.98)	51.01 (13.49)	37.99 (11.96)	43.35 (11.76)	52.24 (15.89)	57.55 (13.49)	60.62 (13.72)	62.94 (11.88)	39.74 (13.16)	45.14 (12.15)
**Death**																
**No. (%) of patients who died during the observation period**	950 (10.71)	2562 (19.50)	918 (10.83)	2461 (19.69)	825 (10.41)	2178 (19.18)	778 (10.43)	2089 (19.12)	74 (12.85)	162 (18.47)	757 (13.94)	2257 (22.65)	343 (34.79)	1154 (42.36)	883 (9.70)	2431 (18.58)
**Age at death, mean (SD), y**	55.18 (17.04)	61.55 (13.59)	55.46 (17.04)	61.85 (13.57)	55.15 (17.38)	61.76 (13.78)	55.39 (17.20)	62.08 (13.62)	45.08 (15.07)	50.31 (12.34)	58.82 (15.87)	63.08 (12.97)	63.62 (14.33)	66.43 (11.68)	53.88 (16.65)	60.76 (13.50)

Abbreviations: OAs, oral antipsychotics; LAIAs, long-acting injectable antipsychotics; ED, emergency department; SD, standard deviation

© 2022 Wei Y et al. *JAMA Network Open.*

eTable 8. Patient Characteristics by Length of Follow-up

Patient characteristics	Individuals prescribed both OAs and LAIAs, with at least one of the following outcome events during the observation period															
	Primary outcomes										Secondary outcomes					
	All-cause ED visits		All-cause hospitalizations		Hospitalizations for psychiatric disorders		Hospitalizations for schizophrenia		Incident suicide attempt		Hospitalizations for somatic disorders		Hospitalizations for cardiovascular diseases		Extrapyramidal symptoms	
	≥ 10 years	< 10 years	≥ 10 years	< 10 years	≥ 10 years	< 10 years	≥ 10 years	< 10 years	≥ 10 years	< 10 years	≥ 10 years	< 10 years	≥ 10 years	< 10 years	≥ 10 years	< 10 years
**No. of patients**	16399	5614	15629	5344	14416	4867	13842	4543	1177	276	11955	3441	2820	890	16484	5698
**Sex, No. (%)**																
**Female**	7873 (48.01)	2678 (47.70)	7542 (48.26)	2568 (48.05)	6959 (48.27)	2358 (48.45)	6667 (48.17)	2192 (48.25)	502 (42.65)	111 (40.22)	5836 (48.82)	1584 (46.03)	1278 (45.32)	375 (42.13)	7887 (47.85)	2759 (48.42)
**Male**	8526 (51.99)	2936 (52.30)	8087 (51.74)	2776 (51.95)	7457 (51.73)	2509 (51.55)	7175 (51.83)	2351 (51.75)	675 (57.35)	165 (59.78)	6119 (51.18)	1857 (53.97)	1542 (54.68)	515 (57.87)	8597 (52.15)	2939 (51.58)
**Age, mean (SD), y**																
**At first schizophrenia diagnosis**	38.15 (11.97)	41.13 (14.78)	38.12 (12.06)	41.36 (14.83)	37.78 (12.11)	40.69 (14.69)	37.87 (12.11)	40.71 (14.74)	33.01 (10.73)	34.62 (11.34)	39.46 (12.38)	44.58 (15.30)	45.71 (12.17)	52.71 (13.82)	38.01 (11.76)	40.60 (14.15)
**At cohort entry**	41.52 (11.93)	42.30 (15.43)	41.50 (12.04)	42.52 (15.48)	41.17 (12.12)	41.74 (15.30)	41.27 (12.13)	41.79 (15.36)	36.10 (10.92)	35.88 (11.84)	42.97 (12.28)	46.18 (15.96)	49.54 (11.65)	55.48 (13.98)	41.36 (11.71)	41.70 (14.73)
**At time of event**	48.20 (13.16)	47.25 (16.51)	51.27 (14.48)	49.59 (16.98)	48.26 (13.94)	45.42 (16.64)	48.90 (13.87)	46.27 (16.77)	41.84 (12.06)	38.62 (12.05)	56.16 (14.01)	55.50 (15.92)	62.11 (11.97)	63.26 (13.56)	43.36 (12.31)	41.56 (14.52)
**Death**																
**No. (%) of patients who died during the observation period**	1501 (9.15)	2011 (35.82)	1470 (9.41)	1909 (35.72)	1384 (9.60)	1619 (33.26)	1341 (9.69)	1526 (33.59)	103 (8.75)	133 (48.19)	1394 (11.66)	1620 (47.08)	801 (28.40)	696 (78.20)	1414 (8.58)	1900 (33.35)
**Age at death, mean (SD), y**	64.30 (12.43)	56.49 (15.66)	64.41 (12.48)	56.81 (15.69)	64.41 (12.57)	56.13 (16.09)	64.51 (12.51)	56.54 (15.94)	55.00 (11.77)	43.76 (12.62)	65.02 (12.26)	59.43 (14.66)	67.40 (11.41)	63.92 (13.18)	63.81 (12.30)	55.30 (15.32)

Abbreviations: OAs, oral antipsychotics; LAIAs, long-acting injectable antipsychotics; ED, emergency department; SD, standard deviation

© 2022 Wei Y et al. *JAMA Network Open.*

eTable 9. Patient Characteristics by Substance Use

Patient characteristics	Individuals prescribed both OAs and LAIAs, with at least one of the following outcome events during the observation period															
	Primary outcomes										Secondary outcomes					
	**All-cause ED visits**		**All-cause hospitalizations**		**Hospitalizations for psychiatric disorders**		**Hospitalizations for schizophrenia**		**Incident suicide attempt**		**Hospitalizations for somatic disorders**		**Hospitalizations for cardiovascular diseases**		**Extrapyramidal symptoms**	
	Without substance use	With substance use	Without substance use	With substance use	Without substance use	With substance use	Without substance use	With substance use	Without substance use	With substance use	Without substance use	With substance use	Without substance use	With substance use	Without substance use	With substance use
**No. of patients**	19816	2197	18819	2154	17189	2094	16490	1895	812	641	13694	1702	3464	246	20020	2162
**Sex, No. (%)**																
**Female**	9859 (49.75)	692 (31.50)	9429 (50.10)	681 (31.62)	8651 (50.33)	666 (31.81)	8254 (50.05)	605 (31.93)	432 (53.20)	181 (28.24)	6869 (50.16)	551 (32.37)	1570 (45.32)	83 (33.74)	9972 (49.81)	674 (31.17)
**Male**	9957 (50.25)	1505 (68.50)	9390 (49.90)	1473 (68.38)	8538 (49.67)	1428 (68.19)	8236 (49.95)	1290 (68.07)	380 (46.80)	460 (71.76)	6825 (49.84)	1151 (67.63)	1894 (54.68)	163 (66.26)	10048 (50.19)	1488 (68.83)
**Age, mean (SD), y**																
**At first schizophrenia diagnosis**	39.71 (12.89)	31.66 (9.38)	39.78 (12.98)	31.62 (9.39)	39.38 (12.98)	31.46 (9.27)	39.40 (12.97)	31.35 (9.22)	35.26 (11.58)	30.85 (9.33)	41.69 (13.25)	31.89 (9.61)	48.11 (12.75)	37.17 (10.98)	39.44 (12.53)	31.63 (9.29)
**At cohort entry**	42.61 (12.95)	33.63 (9.38)	42.69 (13.04)	33.61 (9.40)	42.27 (13.06)	33.46 (9.29)	42.32 (13.06)	33.39 (9.27)	38.23 (11.69)	33.31 (9.61)	44.89 (13.15)	34.05 (9.66)	51.73 (12.26)	40.25 (11.02)	42.29 (12.57)	33.61 (9.28)
**At time of event**	49.68 (13.71)	40.04 (10.39)	52.84 (14.80)	39.84 (10.85)	49.67 (14.59)	38.76 (10.22)	49.93 (14.52)	39.48 (10.32)	43.76 (12.63)	38.02 (10.62)	57.66 (13.86)	42.73 (11.75)	63.05 (12.13)	51.05 (11.04)	43.90 (12.91)	35.70 (9.59)
**Death**																
**No. (%) of patients who died during the observation period**	3218 (16.24)	294 (13.38)	3089 (16.41)	290 (13.46)	2724 (15.85)	279 (13.32)	2626 (15.92)	241 (12.72)	120 (14.78)	116 (18.10)	2764 (20.18)	250 (14.69)	1423 (41.08)	74 (30.08)	3026 (15.11)	288 (13.32)
**Age at death, mean (SD), y**	61.16 (14.35)	45.24 (12.59)	61.51 (14.29)	45.25 (12.56)	61.47 (14.55)	45.01 (12.44)	61.60 (14.47)	45.71 (12.48)	51.64 (13.68)	45.59 (12.53)	63.42 (13.12)	46.43 (12.44)	66.48 (12.00)	52.46 (12.18)	60.25 (14.22)	45.00 (12.54)

Abbreviations: OAs, oral antipsychotics; LAIAs, long-acting injectable antipsychotics; ED, emergency department; SD, standard deviation

© 2022 Wei Y et al. *JAMA Network Open.*

eTable 10. Sensitivity Analysis by Restricting the Age Range of Early/Late LAIAs Initiators

	**Full treatment periods**			
	**Early LAIAs initiators**		**Late LAIAs initiators**	
**Outcome events**	**Adjusted[#] IRR (95% CI)**	**P value**	**Adjusted[#] IRR (95% CI)**	**P value**
All-cause ED visits	0.71 (0.65, 0.78)	<0.001	0.95 (0.89, 1.01)	0.11
All-cause hospitalizations	0.32 (0.28, 0.37)	<0.001	0.51 (0.46, 0.57)	<0.001
Hospitalizations for psychiatric disorders	0.28 (0.24, 0.33)	<0.001	0.45 (0.40, 0.51)	<0.001
Hospitalizations for schizophrenia	0.28 (0.23, 0.33)	<0.001	0.45 (0.39, 0.52)	<0.001
Incident suicide attempt	0.36 (0.15, 0.85)	0.02	0.63 (0.28, 1.39)	0.25
Hospitalizations for somatic disorders	0.64 (0.49, 0.84)	0.001	0.76 (0.63, 0.91)	0.003
Hospitalizations for cardiovascular diseases	1.00 (0.38, 2.61)	0.99	1.11 (0.53, 2.32)	0.78
Extrapyramidal symptoms	0.41 (0.34, 0.49)	<0.001	1.09 (0.95, 1.25)	0.35

	**Subsequent treatment periods***			
	**Early LAIAs initiators**		**Late LAIAs initiators**	
**Outcome events**	**Adjusted[#] IRR (95% CI)**	**P value**	**Adjusted[#] IRR (95% CI)**	**P value**
All-cause ED visits	0.64 (0.57, 0.72)	<0.001	0.94 (0.87, 1.02)	0.13
All-cause hospitalizations	0.31 (0.26, 0.39)	<0.001	0.45 (0.39, 0.52)	<0.001
Hospitalizations for psychiatric disorders	0.28 (0.22, 0.35)	<0.001	0.40 (0.34, 0.48)	<0.001
Hospitalizations for schizophrenia	0.29 (0.23, 0.38)	<0.001	0.40 (0.33, 0.48)	<0.001
Incident suicide attempt	0.31 (0.06, 1.65)	0.17	0.26 (0.06, 1.18)	0.08
Hospitalizations for somatic disorders	0.56 (0.39, 0.82)	0.002	0.68 (0.53, 0.86)	0.002
Hospitalizations for cardiovascular diseases	1.12 (0.47, 2.67)	0.67	1.08 (0.51, 2.29)	0.82
Extrapyramidal symptoms	0.38 (0.24, 0.60)	<0.001	0.52 (0.35, 0.79)	0.002

Note: The method has been described in eMethod – Sensitivity analysis 10.

Abbreviations: IRR, incidence rate ratio; CI, confidence interval; ED, emergency department.

*Beyond the first 90 days in each treatment period

#Adjusted for age (1-year bands) and season (cut by the first date of March, May, September, and November)

© 2022 Wei Y et al. *JAMA Network Open.*

eTable 11. Results From E-Value Analyses

Outcome	Adjusted# IRR (95% CI)	P value	E-value	Upper confidence interval of E-value
All-cause emergency department visits	0.99 (0.97, 1.00)	0.09	1.11	1
All-cause hospitalizations	0.63 (0.61, 0.65)	<0.001	2.55	2.45
Hospitalizations for psychiatric disorders	0.52 (0.50, 0.53)	<0.001	3.26	3.18
Hospitalizations for schizophrenia	0.53 (0.51, 0.55)	<0.001	3.18	3.04
Incident suicide attempt	0.56 (0.44, 0.71)	<0.001	2.97	2.17
Hospitalizations for somatic disorders	0.88 (0.85, 0.91)	<0.001	1.53	1.43
Hospitalizations for cardiovascular diseases	0.88 (0.81, 0.96)	<0.01	1.53	1.25
Extrapyramidal symptoms	0.86 (0.82, 0.91)	<0.001	1.60	1.43

Abbreviations: IRR, incidence rate ratio; CI, confidence interval.

#Adjusted for age (1-year bands) and season (cut by the first date of March, May, September, and November)

In our study, the IRR for all-cause hospitalization was 0.63 with a 95% confidence interval of 0.61 to 0.65. The E-value for the result point estimate was 2.55 with the upper confidence interval being 2.45 in an IRR scale. The results indicate that our observed 0.63-fold decrease in the risk of all-cause hospitalization could be explained by an unmeasured time-varying confounder that was associated with both the treatment and the outcome by a risk ratio of 2.55-fold each, but weaker confounding could not do so. Similar explanations can be applied to other outcomes (except for emergency department visits). Since the confidence interval of emergency department visits includes null (IRR=1), no confounding is needed to move the confidence interval to include 1.

© 2022 Wei Y et al. *JAMA Network Open.*

eTable 12. Results of Self-controlled Case Series Analysis for the Use of LAIAs Alone vs OAs Alone and the Risk of Outcome Events, During the First 90 Days of Treatment

	First 90 days of treatment					
**Outcome event**	**Events, No.**	**Person-years**	**Incidence per 100 person-years**	**Unadjusted IRR (95% CI)**	**Adjusted[#] IRR (95% CI)**	**P value**
**Primary outcomes**						
All-cause ED visits						
OAs alone	25 556	12 743	200.55	Ref	Ref	
LAIAs alone	10 951	5615	195.01	0.88 (0.86, 0.91)	0.89 (0.87, 0.91)	<0.001
All-cause hospitalizations						
OAs alone	15 043	12 264	122.66	Ref	Ref	
LAIAs alone	4133	5374	76.91	0.54 (0.52, 0.56)	0.53 (0.51, 0.55)	<0.001
Hospitalizations for psychiatric disorders						
OAs alone	11 341	11 516	98.48	Ref	Ref	
LAIAs alone	2590	4976	52.06	0.44 (0.42, 0.46)	0.44 (0.42, 0.46)	<0.001
Hospitalizations for schizophrenia						
OAs alone	9142	10 845	84.30	Ref	Ref	
LAIAs alone	2196	4727	46.45	0.45 (0.43, 0.47)	0.45 (0.43, 0.47)	<0.001
Incident suicide attempt						
OAs alone	284	1595	17.80	Ref	Ref	
LAIAs alone	51	619	8.24	0.39 (0.29, 0.54)	0.45 (0.33, 0.62)	<0.001
**Secondary outcomes**						
Hospitalizations for somatic disorders						
OAs alone	5294	8495	62.32	Ref	Ref	
LAIAs alone	2111	3758	56.18	0.81 (0.77, 0.85)	0.81 (0.77, 0.86)	<0.001
Hospitalizations for cardiovascular diseases						
OAs alone	800	2315	34.56	Ref	Ref	
LAIAs alone	362	1078	33.57	0.84 (0.73, 0.96)	0.88 (0.77, 1.01)	0.07
Extrapyramidal symptoms						
OAs alone	11 524	15 861	72.66	Ref	Ref	
LAIAs alone	3559	6518	54.61	0.62 (0.60, 0.65)	0.80 (0.76, 0.84)	<0.001

© 2022 Wei Y et al. *JAMA Network Open.*

eTable 13. Results of Self-controlled Case Series Analysis for the Full Treatment Period of OAs Alone, Full Treatment Period of LAIAs Alone, and Full Treatment Period of the Combination Use of OAs Plus LAIAs vs Baseline Period or Preexposure Period, and the Risk of Outcome Events

**Full treatment period**									
					**Compared with baseline**			**Compared with preexposure period**	
**Outcome event**	**Events, No.**	**Person-years**	**Incidence per 100 person-years**		**Adjusted[#] IRR (95% CI)**	**P value**		**Adjusted[#] IRR (95% CI)**	**P value**
**Primary outcomes**									
All-cause ED visits									
Baseline	20 292	29 988	67.67		Ref	Ref		0.25 (0.24, 0.25)	<0.001
Preexposure to antipsychotics	14 253	3881	367.27		4.05 (3.95, 4.14)	<0.001		Ref	Ref
OAs alone	127 994	104 099	122.95		1.76 (1.72, 1.79)	<0.001		0.43 (0.43, 0.44)	<0.001
LAIAs alone	37 564	29 971	125.33		1.71 (1.67, 1.74)	<0.001		0.42 (0.41, 0.43)	<0.001
Combination use of OAs plus LAIAs	106 522	92 278	115.44		1.78 (1.75, 1.82)	<0.001		0.44 (0.43, 0.45)	<0.001
All-cause hospitalizations									
Baseline	5377	28 786	18.68		Ref	Ref		0.08 (0.07, 0.08)	<0.001
Preexposure to antipsychotics	9715	3738	259.91		13.01 (12.55, 13.49)	<0.001		Ref	Ref
OAs alone	65 377	100 735	64.90		4.05 (3.92, 4.19)	<0.001		0.31 (0.30, 0.32)	<0.001
LAIAs alone	12 592	28 287	44.52		2.51 (2.42, 2.61)	<0.001		0.19 (0.19, 0.20)	<0.001
Combination use of OAs plus LAIAs	40 943	84 615	48.39		3.15 (3.05, 3.27)	<0.001		0.24 (0.24, 0.25)	<0.001
Hospitalizations for psychiatric disorders									
Baseline	1564	26 561	5.89		Ref	Ref		0.03 (0.02, 0.03)	<0.001
Preexposure to antipsychotics	8268	3516	235.16		39.50 (37.29, 41.84)	<0.001		Ref	Ref
OAs alone	40 010	94 317	42.42		10.15 (9.59, 10.74)	<0.001		0.26 (0.25, 0.26)	<0.001
LAIAs alone	6373	2568	24.82		5.13 (4.82, 5.46)	<0.001		0.13 (0.13, 0.13)	<0.001
Combination use of OAs plus LAIAs	23 857	72 893	32.73		7.97 (7.52, 8.44)	<0.001		0.20 (0.20, 0.21)	<0.001
Hospitalizations for schizophrenia									
Baseline	1123	24 484	4.59		Ref	Ref		0.02 (0.02, 0.02)	<0.001
Preexposure to antipsychotics	6837	3295	207.47		47.39 (44.31, 50.67)	<0.001		Ref	Ref
OAs alone	33 436	89 539	37.34		11.41 (10.67, 12.19)	<0.001		0.24 (0.23, 0.25)	<0.001
LAIAs alone	5499	24 372	22.56		5.88 (5.48, 6.32)	<0.001		0.12 (0.12, 0.13)	<0.001
Combination use of OAs plus LAIAs	21 206	70 387	30.13		9.04 (8.45, 9.67)	<0.001		0.19 (0.18, 0.20)	<0.001
Incident suicide attempt									
Baseline	51	2212	2.31		Ref	Ref		0.07 (0.05, 0.10)	<0.001

© 2022 Wei Y et al. *JAMA Network Open.*

Preexposure to antipsychotics	95	453	20.98		14.28 (9.89, 20.62)	<0.001		Ref	Ref
OAs alone	738	8706	8.48		7.60 (5.46, 10.58)	<0.001		0.53 (0.42, 0.67)	<0.001
LAIAs alone	121	2165	5.59		4.26 (2.92, 6.22)	<0.001		0.30 (0.22, 0.40)	<0.001
Combination use of OAs plus LAIAs	448	5781	7.75		7.33 (5.21, 10.32)	<0.001		0.51 (0.40, 0.66)	<0.001
**Secondary outcomes**									
Hospitalizations for somatic disorders									
Baseline	4462	17 739	25.15		Ref	Ref		0.26 (0.25, 0.27)	<0.001
Preexposure to antipsychotics	2385	2498	95.49		3.85 (3.65, 4.06)	<0.001		Ref	Ref
OAs alone	34 526	71 395	48.36		1.99 (1.91, 2.07)	<0.001		0.52 (0.49, 0.54)	<0.001
LAIAs alone	8189	19 745	41.47		1.73 (1.65, 1.82)	<0.001		0.45 (0.43, 0.47)	<0.001
Combination use of OAs plus LAIAs	22 961	63 596	36.1		1.64 (1.57, 1.71)	<0.001		0.43 (0.41, 0.45)	<0.001
Hospitalizations for cardiovascular diseases									
Baseline	726	5124	14.17		Ref	Ref		0.17 (0.15, 0.20)	<0.001
Preexposure to antipsychotics	481	638	75.38		5.78 (5.10, 6.54)	<0.001		Ref	Ref
OAs alone	5746	21830	26.32		2.09 (1.89, 2.32)	<0.001		0.36 (0.33, 0.40)	<0.001
LAIAs alone	1392	6171	22.56		1.83 (1.63, 2.06)	<0.001		0.32 (0.28, 0.36)	<0.001
Combination use of OAs plus LAIAs	3778	2055	18.38		1.73 (1.55, 1.93)	<0.001		0.30 (0.27, 0.33)	<0.001
Extrapyramidal symptoms									
Baseline	462	29 566	1.56		Ref	Ref		0.11 (0.09, 0.12)	<0.001
Preexposure to antipsychotics	384	4252	9.03		9.30 (8.08, 10.71)	<0.001		Ref	Ref
OAs alone	15 889	112 797	14.09		32.68 (29.47, 36.24)	<0.001		3.51 (3.17, 3.90)	<0.001
LAIAs alone	4042	32 482	12.44		28.17 (25.29, 31.38)	<0.001		3.03 (2.72, 3.37)	<0.001
Combination use of OAs plus LAIAs	13 207	101 159	13.06		52.38 (47.18, 58.15)	<0.001		5.63 (5.07, 6.25)	<0.001

Note: The preexposure period was defined as the 30 days before any antipsychotic treatment, and the baseline period was defined as the non-treatment period except the preexposure period.

Abbreviations: OAs, oral antipsychotics; LAIAs, long-acting injectable antipsychotics; IRR, incidence rate ratio; CI, confidence interval; ED, emergency department.

#Adjusted for age (1-year bands) and season (cut by the first date of March, May, September, and November)

© 2022 Wei Y et al. *JAMA Network Open.*

eTable 14. Results of Self-controlled Case Series Analysis for the First 90 Days of OAs Alone, the Subsequent Use of OAs Alone, the First 90 Days of LAIAs Alone, the Subsequent Use of LAIAs Alone, the First 90 Days of OAs plus LAIAs, the Subsequent Use of OAs Plus LAIAs vs Baseline Period or Preexposure Period, and the Risk of Outcome Events

**First 90 days of antipsychotic treatment and the subsequent treatment period***									
					**Compared with baseline**			**Compared with preexposure period**	
**Outcome event**	**Events, No.**	**Person-years**	**Incidence per 100 person-years**		**Adjusted# IRR (95% CI)**	**P value**		**Adjusted# IRR (95% CI)**	**P value**
**Primary outcomes**									
All-cause ED visits									
Baseline	20 292	29 988	67.67		Ref	Ref		0.24 (0.24, 0.25)	<0.001
Preexposure to antipsychotics	14 253	3881	367.27		4.09 (3.99, 4.18)	<0.001		Ref	Ref
First 90 days of OAs alone	25 556	12 743	200.55		2.33 (2.28, 2.38)	<0.001		0.57 (0.56, 0.58)	<0.001
Subsequent use of OAs alone	102 438	91 355	112.13		1.54 (1.51, 1.57)	<0.001		0.38 (0.37, 0.38)	<0.001
First 90 days of LAIAs alone	10 951	5615	195.01		2.04 (1.99, 2.09)	<0.001		0.50 (0.49, 0.51)	<0.001
Subsequent use of LAIAs alone	26 613	24 356	109.27		1.49 (1.46, 1.53)	<0.001		0.37 (0.36, 0.37)	<0.001
First 90 days of OAs plus LAIAs	21 836	11 061	197.41		2.29 (2.24, 2.34)	<0.001		0.56 (0.55, 0.57)	<0.001
Subsequent use of OAs plus LAIAs	84 686	81 217	104.27		1.55 (1.52, 1.58)	<0.001		0.38 (0.37, 0.39)	<0.001
All-cause hospitalizations									
Baseline	5377	28 786	18.68		Ref	Ref		0.08 (0.07, 0.08)	<0.001
Preexposure to antipsychotics	9715	3738	259.91		13.18 (12.72, 13.67)	<0.001		Ref	Ref
First 90 days of OAs alone	15 043	12 264	122.66		6.80 (6.57, 7.05)	<0.001		0.52 (0.50, 0.53)	<0.001
Subsequent use of OAs alone	50 334	88 470	56.89		3.10 (3.00, 3.21)	<0.001		0.24 (0.23, 0.24)	<0.001
First 90 days of LAIAs alone	4133	5374	76.91		3.58 (3.42, 3.74)	<0.001		0.27 (0.26, 0.28)	<0.001
Subsequent use of LAIAs alone	8459	22 913	36.92		1.94 (1.86, 2.02)	<0.001		0.15 (0.14, 0.15)	<0.001
First 90 days of OAs plus LAIAs	10 784	10 421	103.48		5.27 (5.07, 5.47)	<0.001		0.40 (0.39, 0.41)	<0.001
Subsequent use of OAs plus LAIAs	30 159	74 194	40.65		2.35 (2.27, 2.43)	<0.001		0.18 (0.17, 0.18)	<0.001
Hospitalizations for psychiatric disorders									
Baseline	1564	26 561	5.89		Ref	Ref		0.03 (0.02, 0.03)	<0.001
Preexposure to antipsychotics	8268	3516	235.16		39.92 (37.69, 42.28)	<0.001		Ref	Ref
First 90 days of OAs alone	11 341	11 516	98.48		18.59 (17.54, 19.69)	<0.001		0.47 (0.45, 0.48)	<0.001
Subsequent use of OAs alone	28 669	82 801	34.62		7.00 (6.61, 7.41)	<0.001		0.18 (0.17, 0.18)	<0.001
First 90 days of LAIAs alone	2590	4976	52.06		8.07 (7.55, 8.63)	<0.001		0.20 (0.19, 0.21)	<0.001

© 2022 Wei Y et al. *JAMA Network Open.*

Subsequent use of LAIAs alone	3783	20 705	18.27		3.44 (3.22, 3.67)	<0.001		0.09 (0.08, 0.09)	<0.001
First 90 days of OAs plus LAIAs	8156	9454	86.27		14.55 (13.71, 15.44)	<0.001		0.36 (0.35, 0.38)	<0.001
Subsequent use of OAs plus LAIAs	15 701	63 440	24.75		5.13 (4.84, 5.45)	<0.001		0.13 (0.12, 0.13)	<0.001
Hospitalizations for schizophrenia									
Baseline	1123	24 484	4.59		Ref	Ref		0.02 (0.02, 0.02)	<0.001
Preexposure to antipsychotics	6837	3295	207.47		47.85 (44.76, 51.16)	<0.001		Ref	Ref
First 90 days of OAs alone	9142	10 845	84.30		21.43 (20.04, 22.93)	<0.001		0.45 (0.43, 0.46)	<0.001
Subsequent use of OAs alone	24 294	78 694	30.87		7.92 (7.41, 8.47)	<0.001		0.17 (0.16, 0.17)	<0.001
First 90 days of LAIAs alone	2196	4727	46.45		9.46 (8.76, 10.21)	<0.001		0.20 (0.19, 0.21)	<0.001
Subsequent use of LAIAs alone	3303	19 644	16.81		3.95 (3.67, 4.26)	<0.001		0.08 (0.08, 0.09)	<0.001
First 90 days of OAs plus LAIAs	7021	9072	77.39		16.92 (15.79, 18.12)	<0.001		0.35 (0.34, 0.37)	<0.001
Subsequent use of OAs plus LAIAs	14 185	61 314	23.13		5.90 (5.51, 6.31)	<0.001		0.12 (0.12, 0.13)	<0.001
Incident suicide attempt									
Baseline	51	2212	2.31		Ref	Ref		0.07 (0.05, 0.10)	<0.001
Preexposure to antipsychotics	95	453	20.98		14.19 (9.84, 20.45)	<0.001		Ref	Ref
First 90 days of OAs alone	284	1595	17.80		11.72 (8.38, 16.38)	<0.001		0.83 (0.65, 1.05)	0.12
Subsequent use of OAs alone	454	7111	6.38		5.03 (3.59, 7.05)	<0.001		0.35 (0.28, 0.45)	<0.001
First 90 days of LAIAs alone	51	619	8.24		5.25 (3.44, 8.01)	<0.001		0.37 (0.26, 0.53)	<0.001
Subsequent use of LAIAs alone	70	1546	4.53		3.16 (2.08, 4.78)	<0.001		0.22 (0.16, 0.31)	<0.001
First 90 days of OAs plus LAIAs	173	1227	14.10		9.86 (6.93, 14.02)	<0.001		0.69 (0.53, 0.90)	0.007
Subsequent use of OAs plus LAIAs	275	4554	6.04		5.00 (3.51, 7.12)	<0.001		0.35 (0.27, 0.46)	<0.001
**Secondary outcomes**									
Hospitalizations for somatic disorders								0.26 (0.24, 0.27)	<0.001
Baseline	4462	17 739	25.15		Ref	Ref		Ref	Ref
Preexposure to antipsychotics	2385	2498	95.49		3.88 (3.68, 4.09)	<0.001		0.71 (0.67, 0.75)	<0.001
First 90 days of OAs alone	5294	8495	62.32		2.75 (2.62, 2.88)	<0.001		0.45 (0.43, 0.47)	<0.001
Subsequent use of OAs alone	29 232	62 899	46.47		1.75 (1.68, 1.83)	<0.001		0.57 (0.54, 0.61)	<0.001
First 90 days of LAIAs alone	2111	3758	56.18		2.22 (2.09, 2.35)	<0.001		0.39 (0.37, 0.41)	<0.001
Subsequent use of LAIAs alone	6078	15 987	38.02		1.51 (1.44, 1.59)	<0.001		0.54 (0.51, 0.57)	<0.001
First 90 days of OAs plus LAIAs	3668	7618	48.15		2.08 (1.98, 2.20)	<0.001		0.38 (0.36, 0.40)	<0.001
Subsequent use of OAs plus LAIAs	19 293	55 978	34.47		1.48 (1.41, 1.55)	<0.001		0.71 (0.67, 0.75)	<0.001
Hospitalizations for cardiovascular diseases									
Baseline	726	5124	14.17		Ref	Ref		0.17 (0.15, 0.19)	<0.001
Preexposure to antipsychotics	481	638	75.38		5.83 (5.15, 6.61)	<0.001		Ref	Ref

© 2022 Wei Y et al. *JAMA Network Open.*

First 90 days of OAs alone	800	2315	34.56		3.10 (2.75, 3.49)	<0.001		0.53 (0.47, 0.60)	<0.001
Subsequent use of OAs alone	4946	19 516	25.34		1.85 (1.67, 2.06)	<0.001		0.32 (0.29, 0.35)	<0.001
First 90 days of LAIAs alone	362	1078	33.57		2.70 (2.34, 3.12)	<0.001		0.46 (0.40, 0.53)	<0.001
Subsequent use of LAIAs alone	1030	5093	20.22		1.54 (1.35, 1.74)	<0.001		0.26 (0.23, 0.30)	<0.001
First 90 days of OAs plus LAIAs	543	2352	23.08		2.25 (1.97, 2.57)	<0.001		0.38 (0.34, 0.44)	<0.001
Subsequent use of OAs plus LAIAs	3235	18 198	17.78		1.60 (1.43, 1.79)	<0.001		0.27 (0.25, 0.31)	<0.001
Extrapyramidal symptoms									
Baseline	462	29 566	1.56		Ref	Ref		0.12 (0.11, 0.14)	<0.001
Preexposure to antipsychotics	384	4252	9.03		8.28 (7.20, 9.51)	<0.001		Ref	Ref
First 90 days of OAs alone	11 524	15 861	72.66		64.00 (57.94, 70.70)	<0.001		7.73 (6.98, 8.57)	<0.001
Subsequent use of OAs alone	4365	96 937	4.50		5.78 (5.21, 6.41)	<0.001		0.70 (0.63, 0.78)	<0.001
First 90 days of LAIAs alone	3559	6518	54.61		51.06 (46.04, 56.62)	<0.001		6.17 (5.54, 6.86)	<0.001
Subsequent use of LAIAs alone	483	25 964	1.86		2.31 (2.02, 2.65)	<0.001		0.28 (0.24, 0.32)	<0.001
First 90 days of OAs plus LAIAs	11 452	13 903	82.37		90.73 (82.10, 100.26)	<0.001		10.96 (9.88, 12.15)	<0.001
Subsequent use of OAs plus LAIAs	1755	87 256	2.01		3.00 (2.69, 3.36)	<0.001		0.36 (0.32, 0.41)	<0.001

Note: The preexposure period was defined as the 30 days before any antipsychotic treatment and the baseline period was defined as the non-treatment period except the preexposure period.

Abbreviations: OAs, oral antipsychotics; LAIAs, long-acting injectable antipsychotics; IRR, incidence rate ratio; CI, confidence interval; ED, emergency department.

*Beyond the first 90 days in each treatment period

#Adjusted for age (1-year bands) and season (cut by the first date of March, May, September, and November)

© 2022 Wei Y et al. *JAMA Network Open.*

eTable 15. The Rate of Outcome Events During Different Risk Windows

Outcome event	Events, No.	Person-years	Incidence per 100 person-years
**Primary outcomes**			
All-cause ED visits			
Baseline	20 292	29 988	67.67
Preexposure to antipsychotics	14 253	3881	367.27
First 90 days of OAs alone	25 556	12 743	200.55
90-180 days of OAs alone	12 750	8951	142.44
180-365 days of OAs alone	17 797	13 696	129.94
>365 days of OAs alone	71 891	68 708	104.63
First 90 days of LAIAs alone	10 951	5615	195.01
90-180 days of LAIAs alone	4945	3255	151.93
180-365 days of LAIAs alone	6007	4460	134.69
>365 days of LAIAs alone	15 661	16 641	94.11
First 90 days of OAs plus LAIAs	21 836	11 061	197.41
90-180 days of OAs plus LAIAs	10 913	7659	142.49
180-365 days of OAs plus LAIAs	14 594	11 698	124.76
>365 days of OAs plus LAIAs	59 179	61 861	95.67
All-cause hospitalizations			
Baseline	5377	28 786	18.68
Preexposure to antipsychotics	9715	3738	259.91
First 90 days of OAs alone	15 043	12 264	122.66
90-180 days of OAs alone	6380	8656	73.71
180-365 days of OAs alone	8626	13 263	65.04
>365 days of OAs alone	35 328	66 552	53.08
First 90 days of LAIAs alone	4133	5374	76.91
90-180 days of LAIAs alone	1621	3111	52.10
180-365 days of LAIAs alone	1783	4248	41.98
>365 days of LAIAs alone	5055	15 554	32.50
First 90 days of OAs plus LAIAs	10 784	10 421	103.48
90-180 days of OAs plus LAIAs	4156	7230	57.48
180-365 days of OAs plus LAIAs	5246	11 005	47.67
>365 days of OAs plus LAIAs	20 757	55 958	37.09
Hospitalizations for psychiatric disorders			
Baseline	1564	26 561	5.89
Preexposure to antipsychotics	8268	3516	235.16
First 90 days of OAs alone	11 341	11 516	98.48
90-180 days of OAs alone	4101	8152	50.31
180-365 days of OAs alone	5362	12 462	43.03
>365 days of OAs alone	19 206	62 188	30.88
First 90 days of LAIAs alone	2590	4976	52.06
90-180 days of LAIAs alone	934	2875	32.49
180-365 days of LAIAs alone	944	3908	24.15
>365 days of LAIAs alone	1905	13 921	13.68
First 90 days of OAs plus LAIAs	8156	9454	86.27

© 2022 Wei Y et al. *JAMA Network Open.*

90-180 days of OAs plus LAIAs	2628	6525	40.28
180-365 days of OAs plus LAIAs	3189	9856	32.35
>365 days of OAs plus LAIAs	9884	47 059	21.00
Hospitalizations for schizophrenia			
Baseline	1123	24 484	4.59
Preexposure to antipsychotics	6837	3295	207.47
First 90 days of OAs alone	9142	10 845	84.30
90-180 days of OAs alone	3317	7695	43.10
180-365 days of OAs alone	4443	11 784	37.70
>365 days of OAs alone	16 534	59 214	27.92
First 90 days of LAIAs alone	2196	4727	46.45
90-180 days of LAIAs alone	795	2727	29.16
180-365 days of LAIAs alone	804	3702	21.72
>365 days of LAIAs alone	1704	13 215	12.89
First 90 days of OAs plus LAIAs	7021	9072	77.39
90-180 days of OAs plus LAIAs	2293	6270	36.57
180-365 days of OAs plus LAIAs	2807	9495	29.56
>365 days of OAs plus LAIAs	9085	45 549	19.95
Incident suicide attempt			
Baseline	51	2212	2.31
Preexposure to antipsychotics	95	453	20.98
First 90 days of OAs alone	284	1595	17.80
90-180 days of OAs alone	86	92	9.35
180-365 days of OAs alone	95	1279	7.43
>365 days of OAs alone	273	4912	5.56
First 90 days of LAIAs alone	51	619	8.24
90-180 days of LAIAs alone	22	295	7.46
180-365 days of LAIAs alone	20	359	5.58
>365 days of LAIAs alone	28	893	3.14
First 90 days of OAs plus LAIAs	173	1227	14.10
90-180 days of OAs plus LAIAs	51	662	7.70
180-365 days of OAs plus LAIAs	63	879	7.17
>365 days of OAs plus LAIAs	161	3012	5.34
**Secondary outcomes**			
Hospitalizations for somatic disorders			
Baseline	4462	17 739	25.15
Preexposure to antipsychotics	2385	2498	95.49
First 90 days of OAs alone	5294	8495	62.32
90-180 days of OAs alone	3055	6016	50.78
180-365 days of OAs alone	4350	9307	46.74
>365 days of OAs alone	21 827	47 576	45.88
First 90 days of LAIAs alone	2111	3758	56.18
90-180 days of LAIAs alone	921	2174	42.37
180-365 days of LAIAs alone	1142	2983	38.28
>365 days of LAIAs alone	4015	1083	37.07
First 90 days of OAs plus LAIAs	3668	7618	48.15

© 2022 Wei Y et al. *JAMA Network Open.*

90-180 days of OAs plus LAIAs	2037	532	38.29
180-365 days of OAs plus LAIAs	2826	8145	34.70
>365 days of OAs plus LAIAs	14 430	42 513	33.94
Hospitalizations for cardiovascular diseases			
Baseline	726	5124	14.17
Preexposure to antipsychotics	481	638	75.38
First 90 days of OAs alone	800	2315	34.56
90-180 days of OAs alone	471	1728	27.25
180-365 days of OAs alone	719	2783	25.84
>365 days of OAs alone	3756	15 004	25.03
First 90 days of LAIAs alone	362	1078	33.57
90-180 days of LAIAs alone	139	661	21.02
180-365 days of LAIAs alone	177	939	18.85
>365 days of LAIAs alone	714	3493	20.44
First 90 days of OAs plus LAIAs	543	2352	23.08
90-180 days of OAs plus LAIAs	280	1698	16.49
180-365 days of OAs plus LAIAs	427	2653	16.09
>365 days of OAs plus LAIAs	2528	13 846	18.26
Extrapyramidal symptoms			
Baseline	462	29 566	1.56
Preexposure to antipsychotics	384	4252	9.03
First 90 days of OAs alone	11 524	15 861	72.66
90-180 days of OAs alone	841	10 038	8.38
180-365 days of OAs alone	864	14 943	5.78
>365 days of OAs alone	2660	71 956	3.70
First 90 days of LAIAs alone	3559	6518	54.61
90-180 days of LAIAs alone	143	3536	4.04
180-365 days of LAIAs alone	165	4758	3.47
>365 days of LAIAs alone	175	1767	0.99
First 90 days of OAs plus LAIAs	11 452	13 903	82.37
90-180 days of OAs plus LAIAs	478	8491	5.63
180-365 days of OAs plus LAIAs	416	12 647	3.29
>365 days of OAs plus LAIAs	861	66 117	1.30

Abbreviations: OAs, oral antipsychotics; LAIAs, long-acting injectable antipsychotics; ED, emergency department.

© 2022 Wei Y et al. *JAMA Network Open.*

eFigure 1. Illustration of Self-controlled Case Series Study for Indirect Comparison

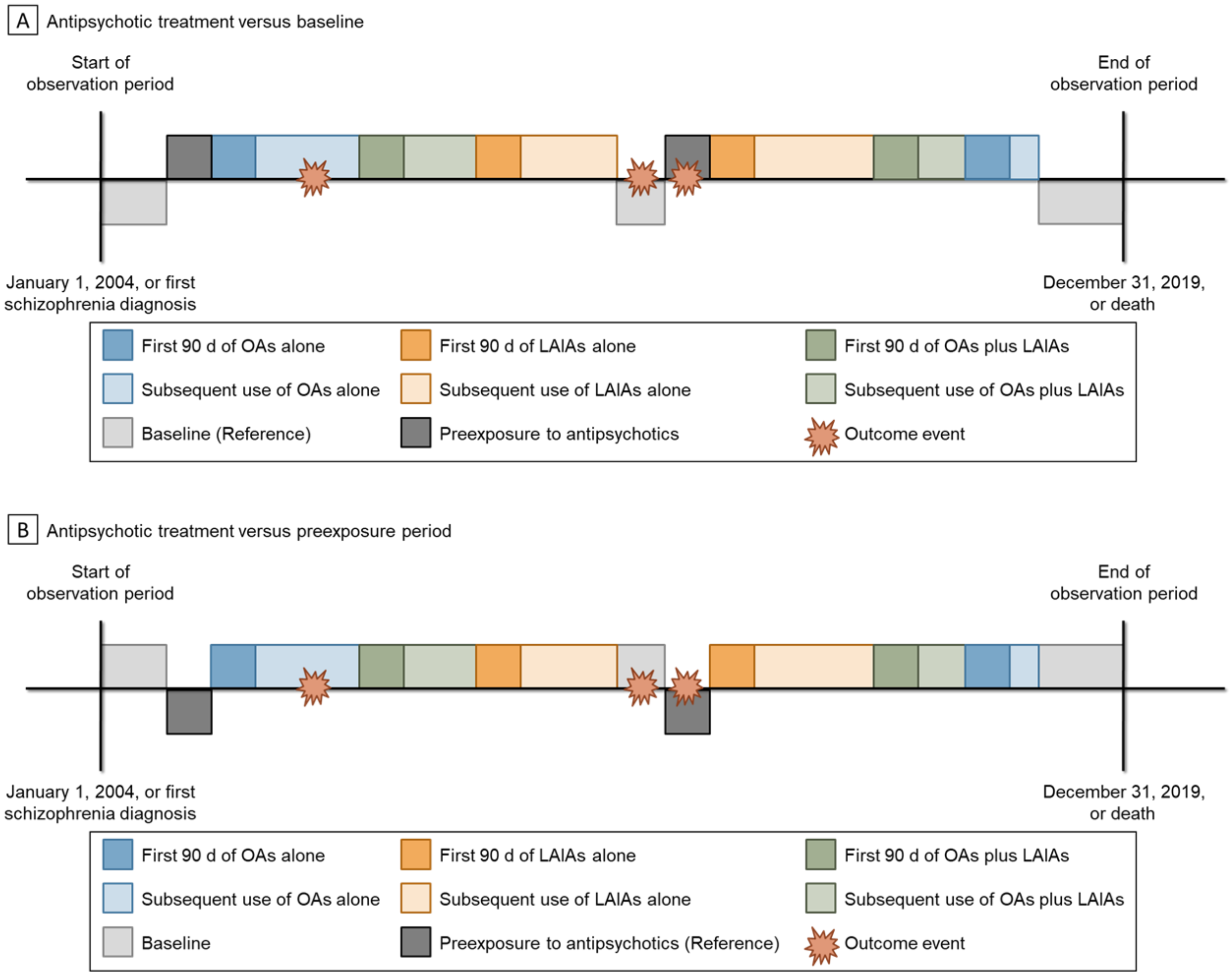

Abbreviations: OAs, oral antipsychotics; LAIAs, long-acting injectable antipsychotics.

Note: This Figure highlights only one example of the potential sequence of medication regimen received, for illustration purposes only.

© 2022 Wei Y et al. *JAMA Network Open.*

eFigure 2. Patient Identification

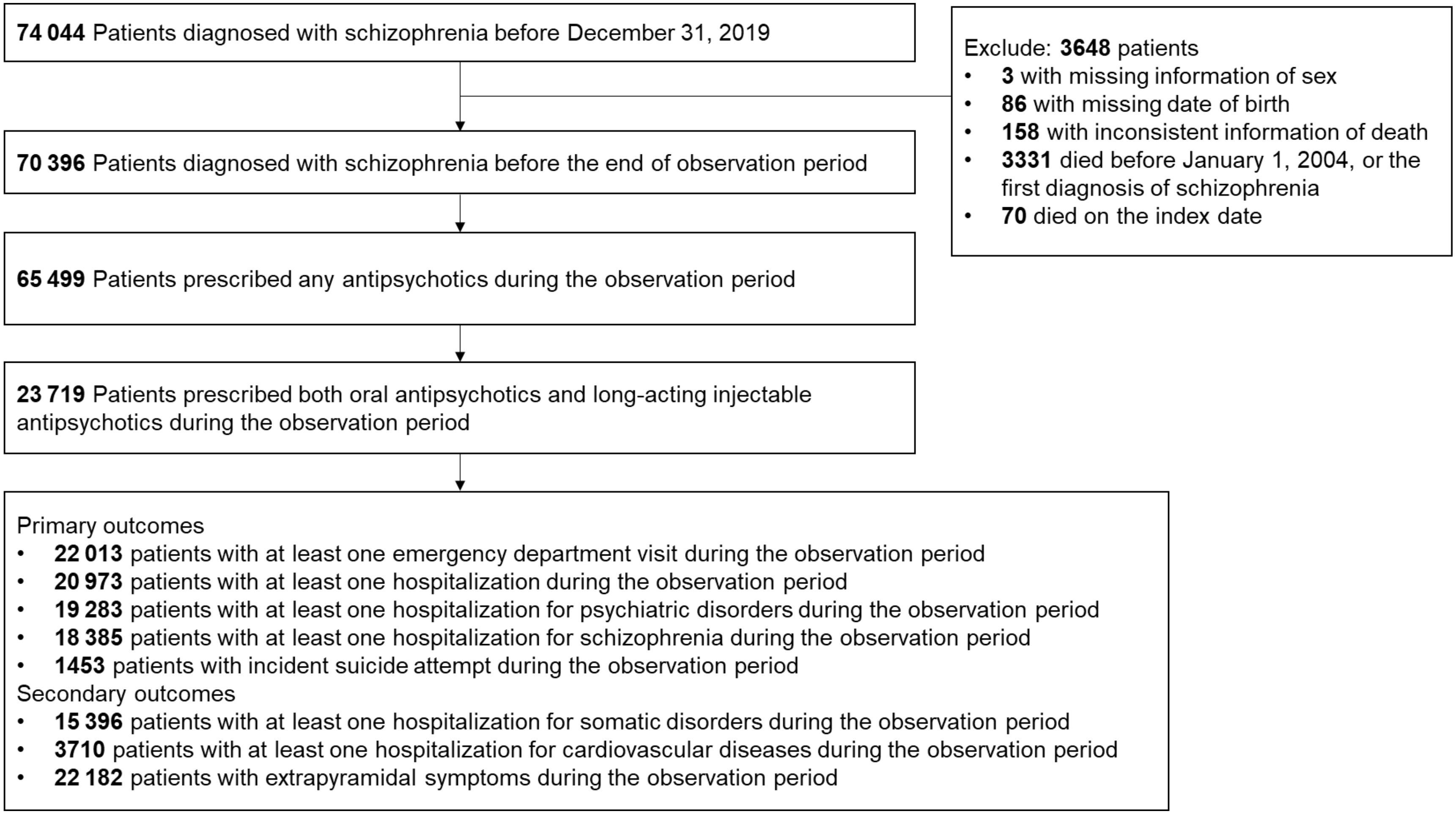

© 2022 Wei Y et al. *JAMA Network Open*.

eFigure 3. Subgroup Analyses for the Use of LAIAs vs OAs and Risk of Outcomes, During the Subsequent Treatment Period

A. Primary outcomes

Subgroups	All-cause emergency department visits IRR (95% CI)	P value	All-cause hospitalizations IRR (95% CI)	P value	Hospitalizations for psychiatric disorders IRR (95% CI)	P value	Hospitalizations for schizophrenia IRR (95% CI)	P value	Incident suicide attempt IRR (95% CI)	P value
**Sex**										
Female	0.95 (0.92, 0.97)	<0.001	0.62 (0.59, 0.65)	<0.001	0.45 (0.42, 0.48)	<0.001	0.46 (0.42, 0.49)	<0.001	0.86 (0.56, 1.32)	0.4846
Male	1.01 (0.98, 1.03)	0.5255	0.65 (0.63, 0.68)	<0.001	0.54 (0.51, 0.58)	<0.001	0.56 (0.52, 0.59)	<0.001	0.48 (0.31, 0.74)	<0.001
**Age, y**										
18-44	1.02 (0.99, 1.05)	0.2279	0.55 (0.52, 0.58)	<0.001	0.47 (0.44, 0.51)	<0.001	0.47 (0.43, 0.50)	<0.001	0.71 (0.47, 1.07)	0.0973
45-65	0.88 (0.86, 0.91)	<0.001	0.61 (0.58, 0.64)	<0.001	0.46 (0.43, 0.49)	<0.001	0.47 (0.44, 0.51)	<0.001	0.68 (0.41, 1.12)	0.1293
> 65	0.94 (0.89, 1.00)	<0.05	0.80 (0.74, 0.86)	<0.001	0.62 (0.55, 0.70)	<0.001	0.64 (0.57, 0.73)	<0.001	0.90 (0.16, 5.14)	0.9038
**Initiation time of LAIAs**										
Early	0.63 (0.58, 0.68)	<0.001	0.39 (0.35, 0.43)	<0.001	0.23 (0.20, 0.27)	<0.001	0.25 (0.22, 0.30)	<0.001	0.58 (0.21, 1.59)	0.2879
Late	0.87 (0.82, 0.91)	<0.001	0.47 (0.43, 0.52)	<0.001	0.37 (0.33, 0.42)	<0.001	0.37 (0.32, 0.42)	<0.001	0.45 (0.21, 1.00)	0.0507
**Schizophrenia cohort**										
Incident cohort	0.85 (0.82, 0.88)	<0.001	0.53 (0.50, 0.56)	<0.001	0.40 (0.37, 0.43)	<0.001	0.39 (0.36, 0.43)	<0.001	0.62 (0.37, 1.04)	0.0685
Prevalent cohort	1.00 (0.98, 1.02)	0.7934	0.70 (0.67, 0.72)	<0.001	0.57 (0.54, 0.60)	<0.001	0.58 (0.55, 0.61)	<0.001	0.64 (0.44, 0.93)	<0.05
**Length of follow-up, y**										
≥ 10	1.00 (0.98, 1.02)	0.9267	0.64 (0.62, 0.66)	<0.001	0.52 (0.50, 0.55)	<0.001	0.53 (0.51, 0.56)	<0.001	0.61 (0.44, 0.85)	<0.01
< 10	0.87 (0.83, 0.92)	<0.001	0.62 (0.57, 0.66)	<0.001	0.41 (0.37, 0.46)	<0.001	0.41 (0.37, 0.47)	<0.001	0.83 (0.36, 1.88)	0.6492
**Substance use**										
Without	0.96 (0.94, 0.98)	<0.001	0.64 (0.62, 0.66)	<0.001	0.49 (0.47, 0.51)	<0.001	0.50 (0.48, 0.52)	<0.001	0.63 (0.42, 0.95)	<0.05
With	1.01 (0.96, 1.06)	0.4952	0.63 (0.58, 0.68)	<0.001	0.56 (0.51, 0.62)	<0.001	0.59 (0.53, 0.66)	<0.001	0.62 (0.39, 0.98)	<0.05

Favors LAIAs | Favors OAs

B. Secondary outcomes

Subgroups	Hospitalizations for somatic disorders IRR (95% CI)	P value	Hospitalizations for cardiovascular diseases IRR (95% CI)	P value	Extrapyramidal symptoms IRR (95% CI)	P value
**Sex**						
Female	0.84 (0.79, 0.89)	<0.001	0.80 (0.69, 0.93)	<0.01	0.38 (0.33, 0.44)	<0.001
Male	0.90 (0.85, 0.96)	<0.001	0.84 (0.73, 0.97)	<0.05	0.42 (0.37, 0.49)	<0.001
**Age, y**						
18-44	0.75 (0.68, 0.82)	<0.001	0.70 (0.48, 1.03)	0.0683	0.33 (0.28, 0.39)	<0.001
45-65	0.83 (0.78, 0.88)	<0.001	0.81 (0.70, 0.95)	<0.01	0.44 (0.38, 0.51)	<0.001
> 65	0.99 (0.92, 1.08)	0.8811	0.88 (0.74, 1.03)	0.1144	0.72 (0.52, 0.98)	<0.05
**Initiation time of LAIAs**						
Early	0.81 (0.69, 0.94)	<0.01	0.88 (0.57, 1.35)	0.5564	0.25 (0.18, 0.34)	<0.001
Late	0.75 (0.66, 0.85)	<0.001	0.62 (0.42, 0.93)	<0.05	0.55 (0.42, 0.73)	<0.001
**Schizophrenia cohort**						
Incident cohort	0.83 (0.77, 0.89)	<0.001	0.71 (0.57, 0.88)	<0.01	0.39 (0.33, 0.46)	<0.001
Prevalent cohort	0.89 (0.85, 0.93)	<0.001	0.88 (0.78, 0.98)	<0.05	0.42 (0.37, 0.48)	<0.001
**Length of follow-up, y**						
≥ 10	0.86 (0.82, 0.90)	<0.001	0.88 (0.79, 0.99)	<0.05	0.41 (0.37, 0.46)	<0.001
< 10	0.97 (0.88, 1.06)	0.4968	0.74 (0.58, 0.95)	<0.05	0.41 (0.32, 0.52)	<0.001
**Substance use**						
Without	0.88 (0.84, 0.92)	<0.001	0.82 (0.74, 0.91)	<0.001	0.40 (0.36, 0.44)	<0.001
With	0.84 (0.75, 0.95)	<0.01	1.07 (0.73, 1.57)	0.7275	0.55 (0.41, 0.74)	< 0.001

Favors LAIAs | Favors OAs

© 2022 Wei Y et al. *JAMA Network Open.*

Abbreviations: OAs, oral antipsychotics; LAIAs, long-acting injectable antipsychotics; IRR, incidence rate ratio; CI, confidence interval; ED, emergency department.

Note: The IRR estimation was adjusted for age (1-year bands) and season (cut by the first date of March, May, September, and November)

© 2022 Wei Y et al. *JAMA Network Open.*

eReferences.

1. Bertolini F, Ostuzzi G, Pievani M, Aguglia A, Bartoli F, Bortolaso P, et al. Comparing long-acting antipsychotic discontinuation rates under ordinary clinical circumstances: A survival analysis from an observational, pragmatic study. *CNS Drugs*. 2021;doi:10.1007/s40263-021-00809-w
2. VanderWeele TJ, Ding P. Sensitivity analysis in observational research: Introducing the e-value. *Ann Intern Med*. 2017;167(4):268-274. doi:10.7326/M16-2607
3. Hirano Y. Risk of extrapyramidal syndromes associated with psychotropic polypharmacy: A study based on large-scale japanese claims data. *Ther Innov Regul Sci*. 2020;54(2):259-268. doi:10.1007/s43441-019-00026-0
4. Abouzaid S, Tian H, Zhou H, Kahler KH, Harris M, Kim E. Economic burden associated with extrapyramidal symptoms in a medicaid population with schizophrenia. *Community Ment Health J*. 2014;50(1):51-8. doi:10.1007/s10597-012-9561-7

© 2022 Wei Y et al. *JAMA Network Open.*
